# Functional interaction between FUS and SMN underlies SMA-like splicing changes in wild-type hFUS mice

**DOI:** 10.1038/s41598-017-02195-0

**Published:** 2017-05-17

**Authors:** Alessia Mirra, Simona Rossi, Silvia Scaricamazza, Michela Di Salvio, Illari Salvatori, Cristiana Valle, Paola Rusmini, Angelo Poletti, Gianluca Cestra, Maria Teresa Carrì, Mauro Cozzolino

**Affiliations:** 1 Fondazione Santa Lucia IRCCS, 00143 Rome, Italy; 2 Dipartimento di Biologia, Università di Roma “Tor Vergata”, Rome, Italy; 3 Istituto di Farmacologia Traslazionale (IFT), CNR, 00133 Rome, Italy; 4 Istituto di Biologia e Patologia Molecolari (IBPM), CNR, 00185 Rome, Italy; 5 Dipartimento di Biologia e Biotecnologia “Charles Darwin”, Università di Roma “Sapienza”, 00185 Rome, Italy; 6 Istituto di Biologia Cellulare e Neurobiologia (IBCN), CNR, 00143 Rome, Italy; 7 Dipartimento di Scienze Farmacologiche e Biomolecolari (DiSFeB), Centro di Eccellenza sulle Malattie Neurodegenerative, Università degli Studi di Milano, 20133 Milan, Italy

## Abstract

Several of the identified genetic factors in Amyotrophic Lateral Sclerosis (ALS) point to dysfunction in RNA processing as a major pathogenic mechanism. However, whether a precise RNA pathway is particularly affected remains unknown. Evidence suggests that FUS, that is mutated in familial ALS, and SMN, the causative factor in Spinal Muscular Atrophy (SMA), cooperate to the same molecular pathway, i.e. regulation of alternative splicing, and that disturbances in SMN-regulated functions, either caused by depletion of SMN protein (as in the case of SMA) or by pathogenic interactions between FUS and SMN (as in the case of ALS) might be a common theme in both diseases. In this work, we followed these leads and tested their pathogenic relevance *in vivo*. FUS-associated ALS recapitulates, in transgenic mice, crucial molecular features that characterise mouse models of SMA, including defects in snRNPs distribution and in the alternative splicing of genes important for motor neurons. Notably, altering SMN levels by haploinsufficiency or overexpression does not impact the phenotypes of mouse or *Drosophila* models of FUS-mediated toxicity. Overall, these findings suggest that FUS and SMN functionally interact and that FUS may act downstream of SMN-regulated snRNP assembly in the regulation of alternative splicing and gene expression.

## Introduction

Amyotrophic Lateral Sclerosis (ALS) and Spinal Muscular Atrophy (SMA) are the most common forms of motor neuron diseases (MNDs). While disease onset, genetic causes and the affected motor neurons vary considerably, SMA and ALS are clearly overlapping, as they are both characterized by the progressive degeneration of motor neurons in the anterior horns of the lumbar spinal cord. Thus, a common pathogenic mechanism shared by the two diseases is possible. However, no obvious clues were available until recently, when mutations in FUS, TDP-43 and C9orf72 genes were described in a large fraction of ALS patients^1^. Since it is now clear that all these genes affect the proper control of ribostasis, alterations in RNA metabolism are widely believed to play a major role in motor neuron degeneration in ALS, similarly to what occurs in SMA. Indeed, *Smn1* gene mutations that are responsible for SMA lead to a pathologic shortage of the protein SMN, a central regulator of small nuclear ribonucleoprotein particle (snRNP) assembly, and thus an indispensable player in RNA post-transcriptional regulation, alternative splicing and mRNA transport^2^.

Mutations in the FUS gene are responsible for a small but significant fraction of familial and sporadic ALS. The features that confer toxicity to these mutations are still quite uncertain. This is testified by the observation that either FUS mis-localization due to mutations in the nuclear localization signal, or the upregulation of wild-type protein expression caused by mutations in its 3′ UTR regulatory region, cause ALS in patients and in animal models^3^, through both gain and loss of function mechanisms^4–6^.

We and others have recently shown that FUS and SMN physically interact, and that pathogenic FUS mutations sequester SMN into cytosolic aggregates thus changing its subcellular distribution in cultured neuronal cells as well as in cells from patients^5,7–9^. Moreover, FUS binds, in addition to SMN, to the spliceosomal snRNPs, and the mis-localized mutant FUS proteins retain a portion of these snRNPs in the cytoplasm, decreasing their effective concentration in the nucleus, and eventually affecting alternative splicing^8,10,11^. This is strikingly similar to what occurs in SMA patients or in mouse models for the disease, where a reduction in the steady-state levels of snRNPs^12,13^, as a consequence of SMN shortage, causes spliceosomal dysfunctions. This strongly suggests not only that FUS mutations and SMN depletion alter the same pathway of snRNP assembly and distribution, reducing the active pool of snRNPs available for the splicing machinery, but also that a functional deficiency in SMN might be involved in FUS-mediated ALS.

Even though the nature of the selective vulnerability of motor neurons in SMA and ALS is still obscure, snRNP dysfunctions have been shown to be more prominent in motor neurons compared to other cell types^14^. Further, it has been proposed that different subtypes of spliceosomal snRNPs might be preferentially affected. In particular, spliceosomal snRNPs that target mRNAs that contain minor introns (known as U12-type introns) have been indicated as to be primary involved^15,16^, and specific U12-type mRNAs have been proposed as to have a crucial role in the pathogenic mechanisms of SMA^16,17^. Although the preferential contribution of U12-regulated splicing in the specific vulnerability of motor neurons in SMA is still highly debated^18^, all these observations support the concept that a general splicing deficit can trigger cell type specific changes targeting a restricted number of genes with paramount importance for motor neuron function. Some of these genes have been recently identified on the basis of their early alterations during SMA progression and their specific contribution to motor neuron function in animal models^16–20^. On these grounds, it is plausible that similar defects in SMN-regulated snRNP activity might contribute to the changes in alternative splicing that have been repeatedly described in FUS-related ALS models, and that alterations in a similar set of target genes might explain the overlapping features of both diseases.

To verify these hypotheses, and to extend our previous observations to an *in vivo* model of FUS-related ALS, transgenic mice overexpressing human wild-type FUS (*hFUS*^+/+^ mice), which develop a severe motor neuron degeneration^21^, were systematically evaluated for the presence of molecular hallmarks of SMA. In addition, *hFUS*^+/+^ mice on an *Smn* deficient background were generated to investigate whether reduced SMN levels could modify the disease course. Finally, the effects of overexpression or downregulation of SMN on the degeneration of *Drosophila* eyes, which is induced by wild-type FUS overexpression, were analysed.

## Results

### Wild-type human FUS does not affect snRNP composition and assembly in transgenic mice spinal cord

Overexpression of wild-type human FUS causes ALS-like phenotypes and early lethality in mice^21^. As a model of FUS-mediated neurodegeneration, homozygous human wild-type FUS (*hFUS*^+/+^) overexpressing mice were therefore used in this study. As expected, the amount of transgenic FUS in these animals is proportional to the copy number of the transgene (Supplementary Figure 1). We analysed *hFUS*^+/+^ animals, as well as control non transgenic (*hFUS*^−/−^) and heterozygous transgenic (*hFUS*^+/−^) mice, for the appearance of key disease phenotypes. While *hFUS*^−/−^ and *hFUS*^+/−^ mice appear normal for the entire time frame considered, *hFUS*^+/+^ mice show clear signs of motor phenotypes and ALS pathology, including a decreased number of motor neurons in the lumbar spinal cord, increased expression of astroglial and microglial inflammatory markers, such as GFAP and Iba1, inability to gain weight, signs of progressive hind limb paralysis and early lethality (Supplementary Figure 1).

**Figure 1:**
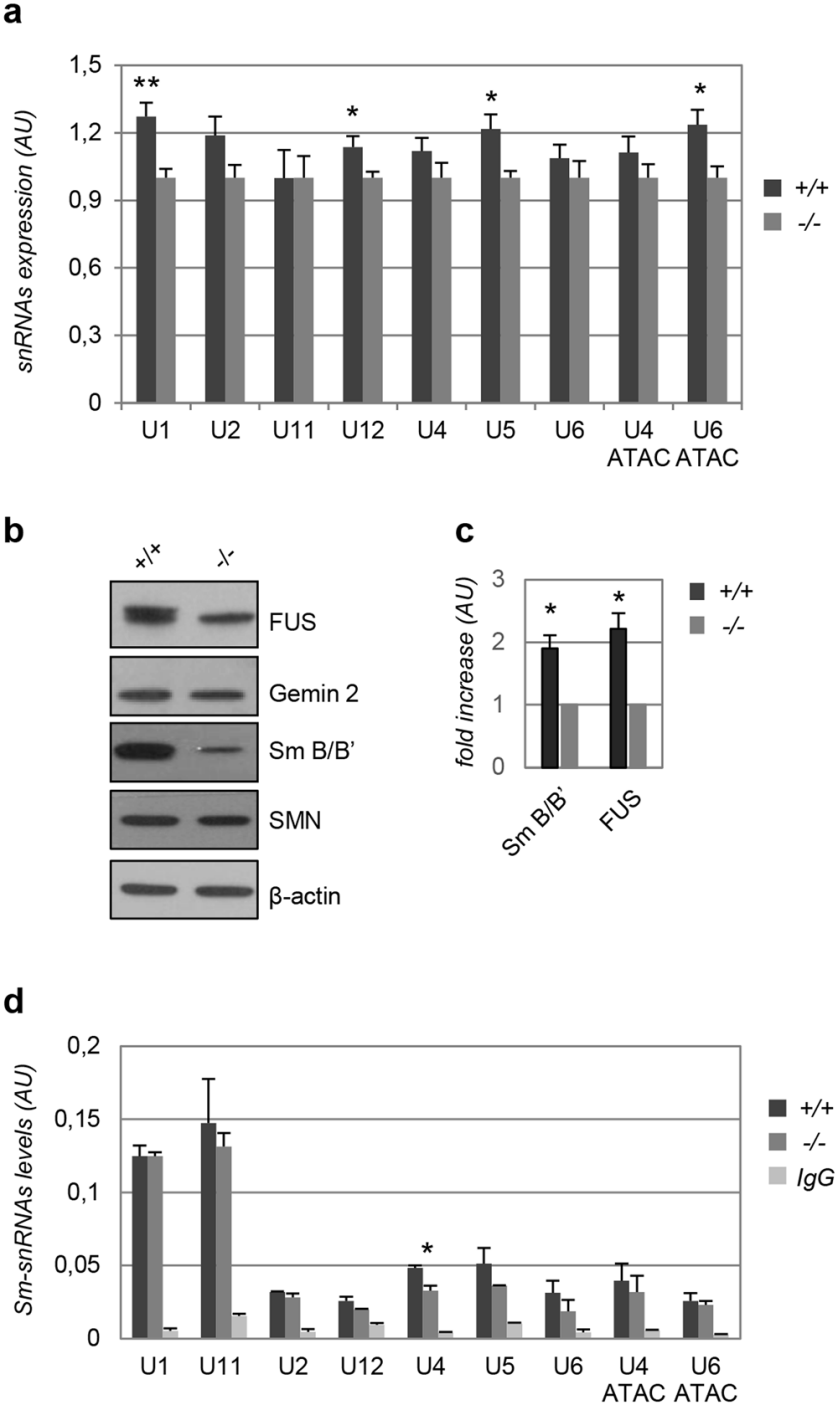
SnRNP composition and assembly in mice spinal cords are not significantly affected by hFUS overexpression. (**a**) RT-qPCR quantification of the indicated snRNAs in lysates from spinal cords of end-stage *hFUS*^+/+^ mice. Means ± SE were normalized for the snRNA levels in control *hFUS*^−/−^ mice. At least 6 animals for each genotype were used. Values significantly different from relative controls are indicated with an asterisk when P ≤ 0.05, and two asterisks when P ≤ 0.01. (**b**) Expression levels of Gemin2, Sm B/B’, SMN, FUS and β-actin were assayed by Western blot on spinal cord lysates from 40 days-old mice of the indicated genotypes. The panel is representative of at least n = 4 independent experiments. Full-length blots are presented in Supplementary Figure 6. (**c**) The expression levels of FUS (endogenous and exogenous) and Sm B/B’ in *hFUS*^+/+^ mice were calculated by densitometric analysis of the bands from Western blots as in (**b**), normalised to β-actin levels and expressed as fold increases over the *hFUS*^−/−^ control animals. Means ± SD are shown from n = 4 independent experiments. Values significantly different from relative controls are indicated with an asterisk when P ≤ 0.05. (**d**) RT-qPCR quantification of snRNAs in the Y12 anti-Sm immunoprecipitates from extracts of *hFUS*^−/−^ and *hFUS*^+/+^ mice. Unrelated IgGs were used in control immunoprecipitates. Values were normalized for the RNA in the input extracts and are reported as mean ± SD. Values significantly different from relative controls are indicated with an asterisk when P ≤ 0.05, and two asterisks when P ≤ 0.01.

To address whether the motor phenotypes of *hFUS*^+/+^ transgenic animals are associated to alterations in snRNPs biogenesis and composition, we analysed the expression of U1, U2, U4, U5, U6, U11, U12, U4atac and U6atac snRNAs by RT-qPCR in spinal cords of animals at late stages of the disease. As shown in Fig. 1a, a slight increase in the expression levels of a vast majority of these snRNAs is observed in diseased animals, reaching statistical significance for U1, U12, U5 and U6atac.

We next analysed the expression of different components of the SMN complex, that is strongly affected in SMA. To this aim, we isolated spinal cords from normal (*hFUS*^−/−^) and diseased (*hFUS*^+/+^) mice and total protein extracts from these tissues were analysed by Western blot with antibodies against SMN, SmB/B’ and Gemin2. As shown in Fig. 1b,c no differences are observed in the expression of SMN and Gemin2, while a twofold increase in the expression of SmB/B’ is detectable in hFUS transgenic animals.

We then immunoprecipitated the Sm core of snRNP complexes from spinal cord extracts using the Y12 antibody that specifically recognizes Sm proteins when assembled into Sm-RNA complexes^22^. As expected, the Y12 antibody, but not control IgGs, specifically co-precipitates the totality of snRNAs analysed, although to different extents (Fig. 1d). However, no major differences in the amount of co-precipitated snRNAs are observed between control and *hFUS*^+/+^ mice, with the exception of U4 snRNA, which shows a modest increased association to the Sm core of the spliceosome in affected animals. From these experiments, we conclude that motor neuron degeneration associated to wild-type hFUS expression in mice is linked to small variations in the expression of the snRNPs components, but not to a major impairment in their assembly into Sm core particles.

It has been described that overexpressed wild-type human FUS is to some extent mislocalised and accumulated into cytosolic granular inclusions in mice motor neurons^21^. We therefore checked whether these inclusions form and whether they contain components of the snRNPs complexes. To this aim, spinal cord sections from control and transgenic mice were assayed by immunofluorescence staining with anti-Sm (Y12), anti-FUS and anti-ChAT antibodies to unequivocally identify motor neurons. As shown in Supplementary Figure 2, no clear cytosolic FUS was observed into motor neurons of transgenic mice. Similarly, cytoplasmic staining of Y12 was hardly detectable, while the antibody, that shows a strong selectivity for the assembled Sm, recognized nuclear dots corresponding to assembled snRNPs.

### Transgenic wild-type hFUS affects the distribution of snRNP complexes in spinal motor neurons

**Figure 2:**
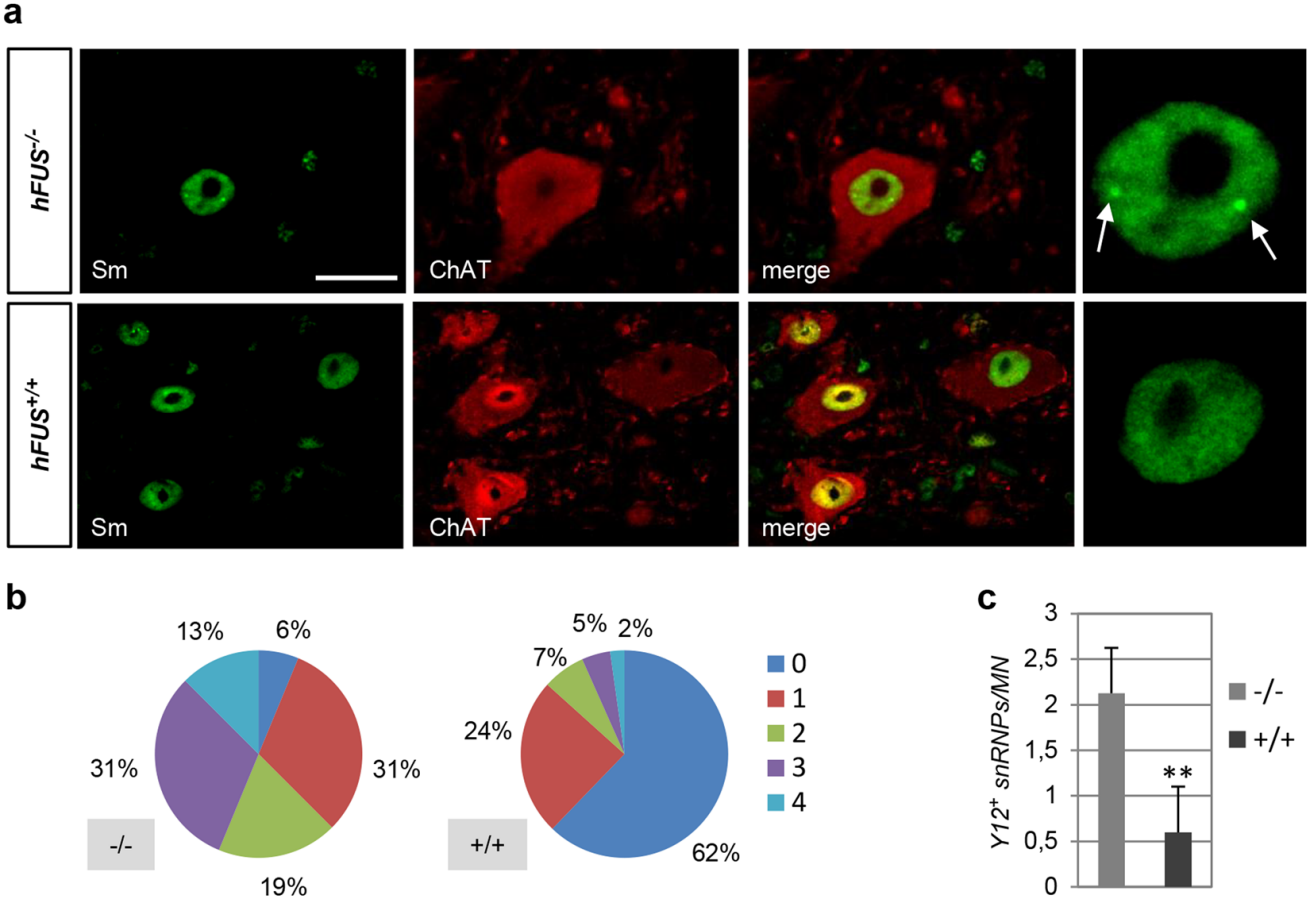
hFUS^+/+^ mice show decreased number of mature snRNPs. (**a**) Lumbar spinal cords from *hFUS*^−/−^ and *hFUS*^+/+^ mice (40 days) were stained with the Y12 antibody to detect Sm proteins and with an anti-ChAT antibody to detect motor neurons. Magnifications are show to highlight the presence of Y12-positive nuclear dots (arrows) corresponding to mature snRNPs. Scale bar: 20 μm. (**b**) Distribution of motor neurons (%) classified according to the number of Y12-positive (Y12+) snRNPs per nucleus. (**c**) Mature snRNPs per motor neuron were scored and the means ± SD are reported. At least 100 motor neurons for each genotype were counted.

To investigate whether the nuclear distribution of snRNPs is affected in motor neurons from *hFUS*^+/+^ transgenic animals, immunofluorescence staining was performed with the Y12 antibody, that recognizes assembled mature snRNPs. In particular, Y12 staining was used to quantify the number of snRNP complexes that are assembled into nuclear dots, in motor neurons from control and diseased animals (Fig. 2a). As shown in Fig. 2b, in control animals the vast majority (81%) of ChAT-positive motor neurons contains 1–3 snRNP complexes, with only a small fraction (6%) that does not display any of these structures. Conversely, the number of motor neurons with no Y12-positive nuclear assemblies rises to 62% in *hFUS*^+/+^ animals (Fig. 2b). These results are confirmed by the analysis reported in Fig. 2c, which shows a significant decrease in the average number of nuclear dots *per* motor neuron in transgenic animals. These findings thus indicate that a defect in the nuclear localization of mature snRNPs characterizes motor neurons of transgenic mice expressing human wild-type FUS. Since no major reduction in the levels of expression of snRNAs, nor in their binding to snRNPs is observed, the net decrease in the number of nuclear snRNPs might reflect a reduced supply of snRNPs to be assembled in nuclear bodies.

### SMA-related alternative splicing events are reproduced in *hFUS*^+/+^ transgenic mice

Following the lead that FUS-linked ALS might share a common disease mechanism with SMA, we then analysed the efficiency of splicing of selected mRNAs that were found altered in SMA mice and tissues from SMA patients, and that might be critical for motor neuron function. In particular, we analysed a set of U12 intron-containing genes, that were shown to be sensitive to SMN deficiency in mammalian SMA cells, including patient cells (Atxn10, Mapk8, Parp1, Vps16, C19orf54, Thoc2, Clcn7, Harsl, Tmem41b)^16,17^. Further, we monitored possible alternative splicing changes in genes that were specifically altered in SMA mice according to transcriptome profiling and that include Mark2, CamK2, Dusp22, Adarb1, Mphosph9, Agrn, Atxn2, and Uspl1^13^.

**Figure 3:**
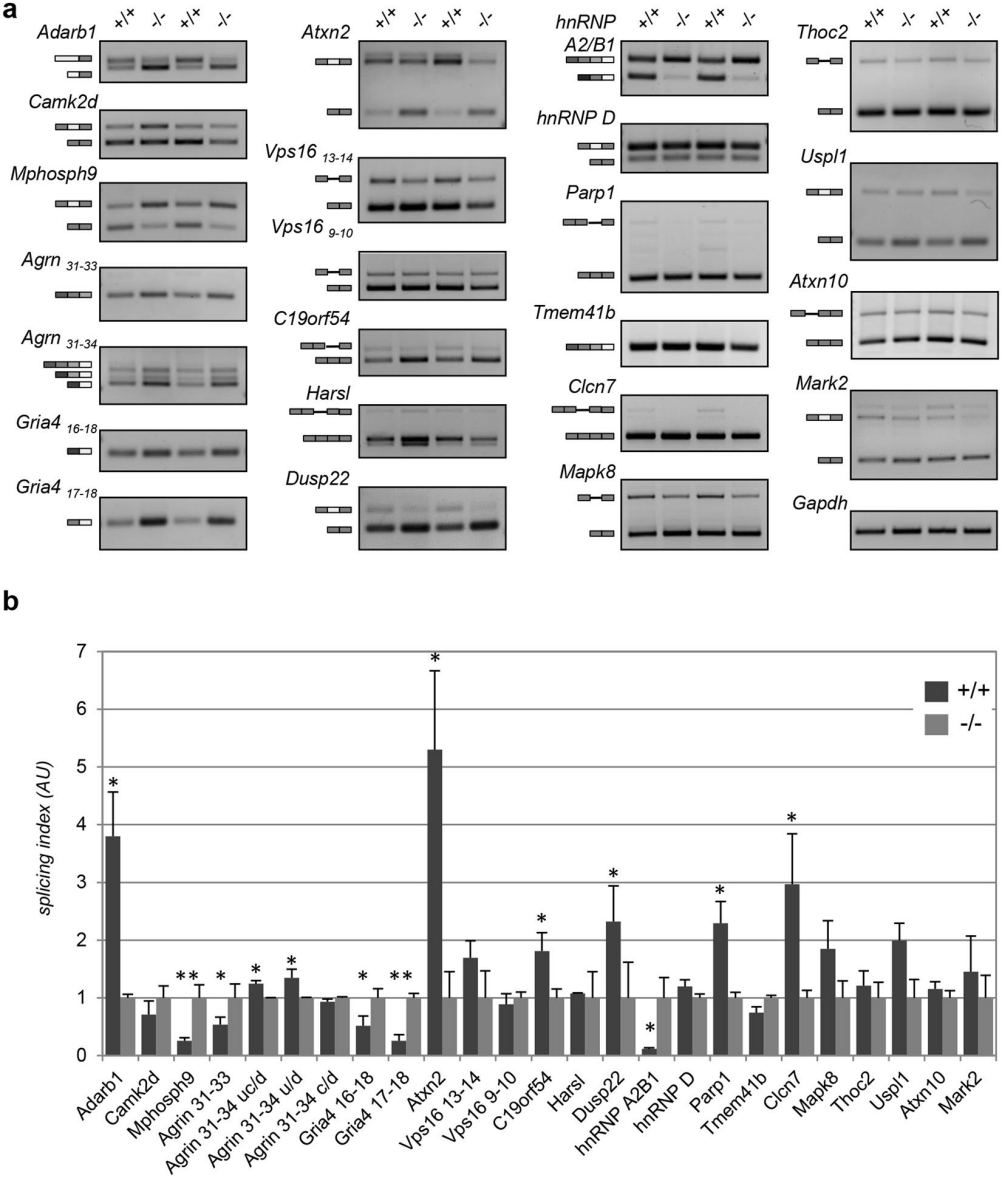
Alternative splicing of SMA target genes is altered in hFUS^+/+^ mice. (**a**) Alternative splicing pattern of selected SMA target exons in *hFUS*^+/+^ and *hFUS*^−/−^ mice. Spinal cords from two different end stage *FUS*^+/+^ mice were analysed, together with age-matched control animals. Results are representative of at least n = 3 independent experiments with 5 different mice. The exons analysed for each gene are reported in Supplemental Table 3. For Gria4, Vps16, and Agrin, different exons were examined, as indicated in subscripts. Schematics of exons (rectangles) and introns (lines) analysed are shown on the left. Full-length agarose gels are presented in Supplementary Figure 7. (**b**) Bands from the experiments exemplified in Fig. 3a were quantified by densitometry analysis, and a splicing index was calculated as follows: for genes that are expressed in more than one isoform, depending on the splicing event involved (i.e. exon skipping/intron retention), the ratios between the upper and the lower bands were calculated and scaled to have the average ratio in *hFUS*^−/−^ mice at 1. For the Agrin gene (exon 31–34), the upper (u), central (**c**) and lower (**d**) bands correspond to isoforms containing exon 31/32/33/34, 31/33/34 and 31/34, respectively. The ratio between bands c/d, u/d and uc/d were calculated as indicated. For genes that are expressed as a unique isoform, the splicing index was calculated as the ratio between band intensity and the relative intensity of the housekeeping Gapdh mRNA. Results from n = 5 independent mice have been considered for each genotype. Mean ± SD is shown. One asterisk is shown when p ≤ 0.05, two asterisks when p ≤ 0.01.

Out of 23 measured splicing events, 12 are significantly affected in *hFUS*^+/+^ transgenic mice at the end-stage of the disease (around 40 days of age; Fig. 3 and Supplementary Table 1). Some of them deserve particular attention, given their critical role for motor neurons function: Gria4, that encodes a core subunit of the AMPA-type glutamate receptor, essential for excitatory synapses and that is affected during ALS progression; Adarb1, which encodes for the ADAR2 enzyme that edits glutamate receptor subunit B pre-mRNA by site-specific deamination of adenosines; Agrin, an heparan sulfate proteoglycan that is required for the development of postsynaptic specializations at the neuromuscular junction. Further, the splicing pattern of two genes that are clearly associated to ALS, such as hnRNP A2/B1 and Atxn2, is also significantly affected.

**Figure 4:**
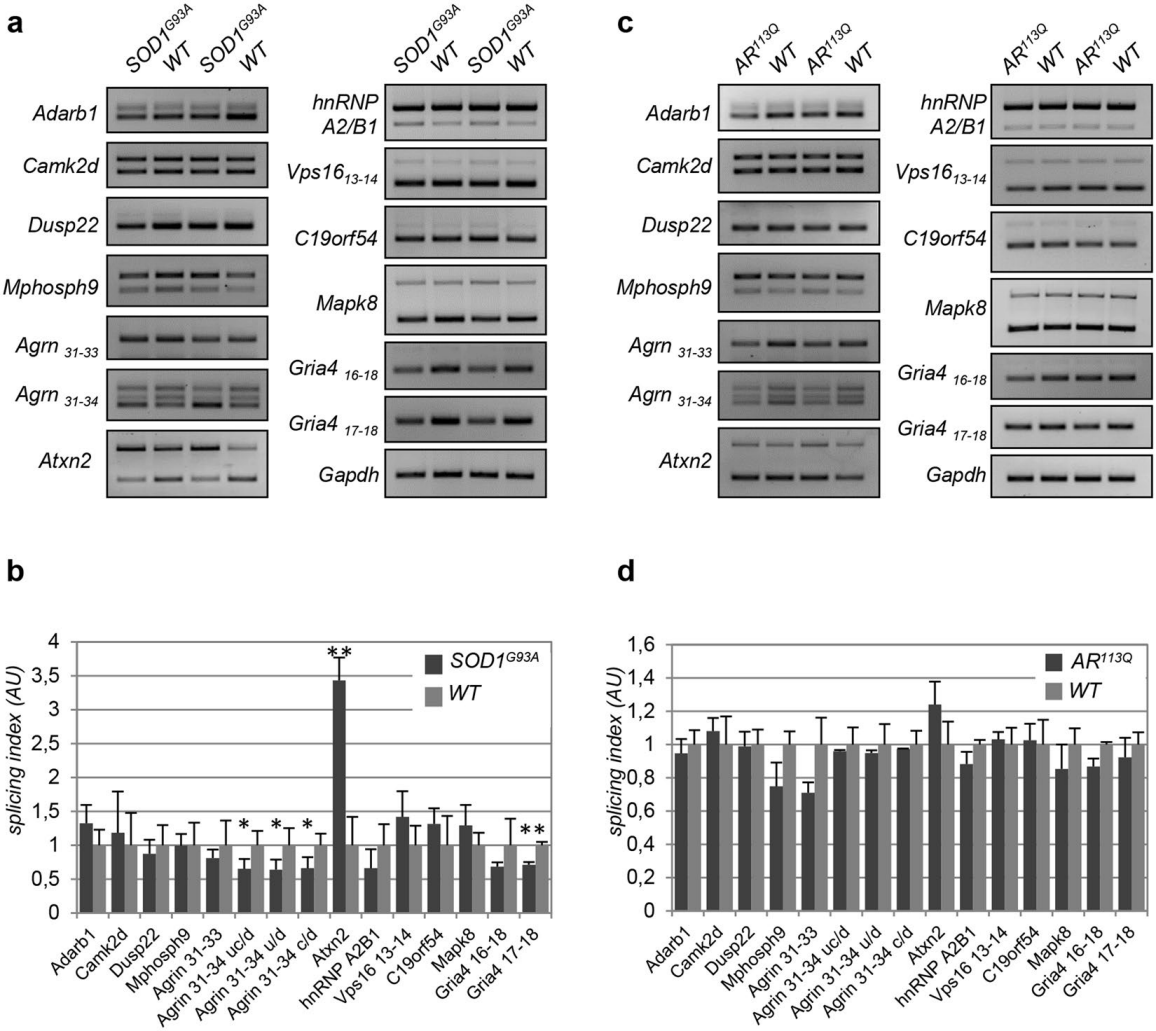
Alternative splicing of SMA target genes is not significantly affected in the spinal cords of SOD1^G93A^-ALS and SBMA mouse models. (**a**) The alternative splicing patterns of selected target exons in the spinal cords of 163 days-old control (WT) and G93A-SOD1 transgenic (*SOD1*^*G93A*^) mice were analysed. Spinal cords from two different mice for each genotype are shown. Results are representative of at least n = 3 independent experiments with 5 different mice. Full-length agarose gels are presented in Supplementary Figure 8. (**b**) Quantification of data in a was performed as explained in the legend of Fig. 3. (**c**) Spinal cords from six months-old male non-transgenic (WT) or knock-in mice expressing an expanded CAG repeat in the endogenous androgen receptor (*AR*^*113Q*^) were analysed for alternative splicing changes in the indicated exons. Full-length agarose gels are presented in Supplementary Figure 8. (**d**) Quantification of data in c was performed as explained and reported as mean ± SD. One asterisk is shown when p ≤ 0.05, two asterisks when p ≤ 0.01.

To exclude that these changes are merely a consequence of the degenerative process occurring in the spinal cord of *hFUS*^+/+^ mice rather than a cause of the degeneration itself, we analysed the alternative splicing of these specific pre-mRNAs in two other mouse models of motor neuron degeneration: the well-established G93A mouse model of ALS, and a Spinal and Bulbar Muscular Atrophy (SBMA) mouse model, where part of the human exon 1 Androgen Receptor gene carrying 113 CAG repeats had been inserted into the homolog region of the endogenous mouse gene^23^. In both cases, spinal cords from diseased, end-stage mice were considered. As shown in Fig. 4a,b, the splicing of Atxn2, Agrin and Gria4 is significantly modified in G93A mice compared to age-matched, control mice. Importantly, however, the splicing of most of the genes that are affected in end-stage *hFUS*^+/+^ mice is not modified. Similarly, no changes in SBMA mice spinal cords are observed in any of the genes taken under consideration (Fig. 4c,d). Overall, these results indicate that specific splicing changes occur during the neurodegenerative process induced in mice spinal cords by human wild-type FUS overexpression, and that these changes overlap those observed in SMA motor neurons.

### SMN deficiency does not modify the disease course in *hFUS*^+/+^ mice

**Figure 5:**
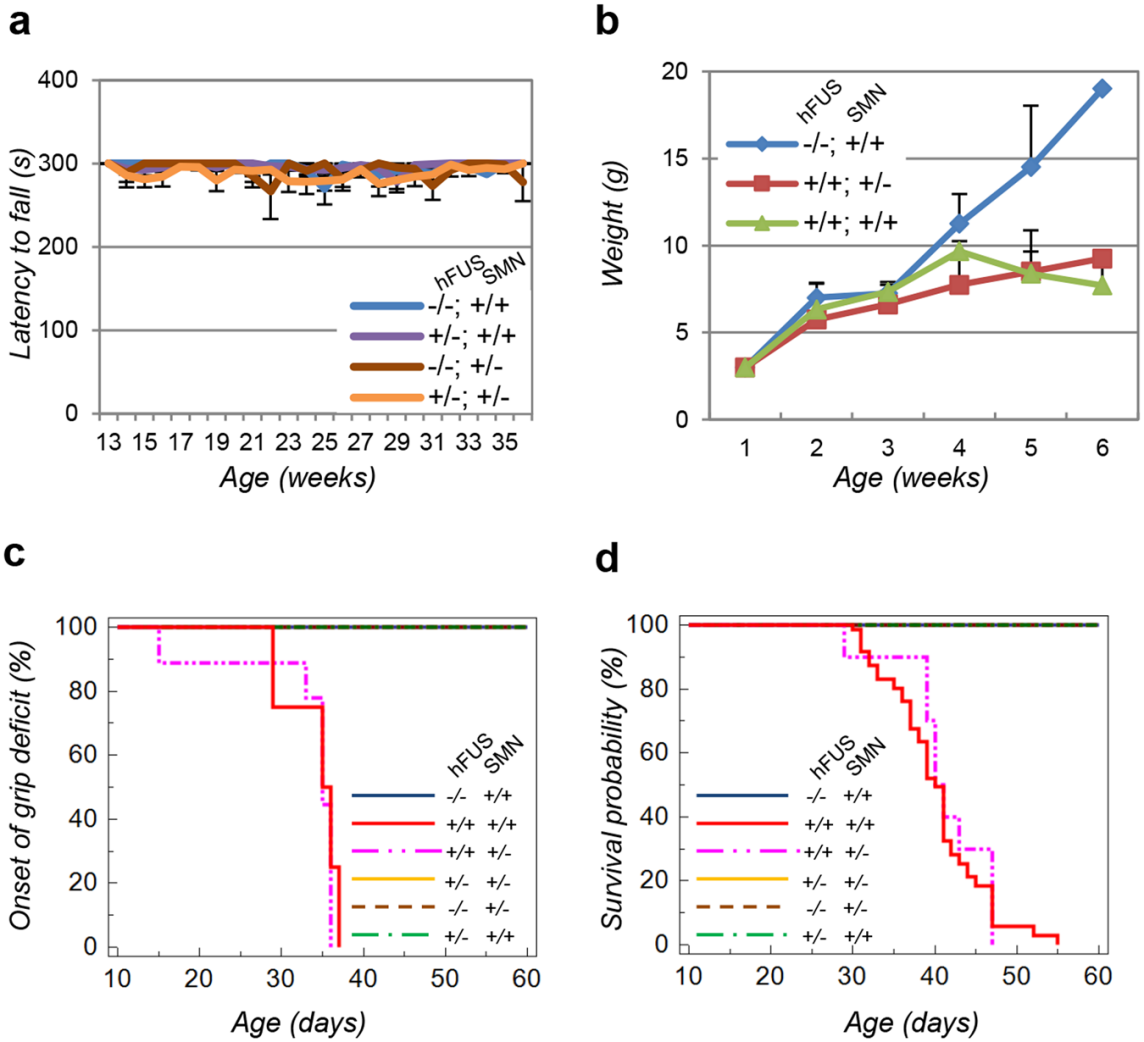
Disease progression in *hFUS*^+/+^ mice is not influenced by SMN depletion. (**a**) The indicated mice were subjected to a Rotarod analysis once a week starting from 13 weeks and for the following 23 weeks. At least 15 animals for each genotype were used. Results were plotted as mean ± SE. (**b**) Mice with the indicated genotypes were weighted weekly starting from 1 week of age. Approximately 5 animals for each genotype were monitored. The average weight ± SD is shown. (**c**) Mice were subjected to grip test analysis for the indicated time frame. The age (days) when animals failed the test were considered the onset of grip deficits and analysed with Kaplan-Meier analysis. Statistical analysis reveals an average onset of grip deficits at 35 ± 4 days for *hFUS*^+/+^; *Smn*^+/+^ mice and at 35 ± 3 days for *hFUS*^+/+^; *Smn*^+/−^ mice. (**d**) Kaplan-Meier analysis of cumulative survival of mice with the indicated genotypes. Log–rank test was used to compare *hFUS*^+/+^; *Smn*^+/−^ and *hFUS*^+/+^; *Smn*^+/+^ animals. At least 30 animals for each genotype were scored, except for the *hFUS*^+/+^; *Smn*^+/−^, where 17 animals were used. Statistical analysis of cumulative survival reveals an average survival time of 40.2 ± 5.8 days for *hFUS*^+/+^; *Smn*^+/+^ mice and 40.6 ± 5.3 days for *hFUS*^+/+^; *Smn*^+/−^ mice.

Results described above support the concept that the pathogenic mechanisms that cause FUS-related ALS and SMA might converge on the mis-splicing of selected, common genes whose expression is crucial for motor neuron viability. Further, they also suggest that defects in the activity of snRNPs, as a consequence of SMN dysfunction, might underlie this process in hFUS transgenic mice. To address this possibility, we generated transgenic *hFUS*^+/+^ mice on an *Smn* deficient background. To this aim, *hFUS*^+/−^ heterozygous mice were crossed with heterozygous *Smn*^+/−^ knock-out mice, that are viable and do not show, in the time frame used for this study, any motor neuron phenotype^24^. The resulting *hFUS*^+/−^; *Smn*^+/−^ mice were then used to produce *hFUS*^+/+^; *Smn*^+/−^ animals. In both cases, as expected, half of SMN levels, compared to control animals, are expressed (Supplementary Figure 3). In these animals, we analysed the effect of SMN gene dosage reduction on motor neuronal phenotypes of hFUS hemizygous mice and hFUS homozygous mice. In particular, we evaluated the motor performance of *hFUS*^+/−^; *Smn*^+/−^ mice in a Rotarod test compared to control (*hFUS*^−/−^; *Smn*^+/+^, *hFUS*^−/−^; *Smn*^+/−^ and *hFUS*^+/^; *Smn*^+/+^) genotypes. As shown in Fig. 5a, no significant variations in motor performances are recorded between the animals analysed, thus indicating that a reduction in *Smn* gene dosage is not sufficient to induce the appearance of motor impairment.

*hFUS*^+/+^ homozygous mice were then analysed for weight loss, motor performance and lifespan. As shown in Fig. 5b, *hFUS*^−/−^ mice gain weight from birth until adulthood, when they reach a plateau. Conversely, *hFUS*^+/+^ animals, independently from the levels of SMN, modestly increase their weight up to around 30–32 days, when they start to progressively lose weight until death. Further, the ability of *hFUS*^+/+^ mice to grip on an inverted mesh grid is significantly impaired at around 35 days of age, and this performance is not affected by decreased levels of SMN in *hFUS*^+/+^; *Smn*^+/−^ mice (Fig. 5c). Given the crucial role of SMN during the development of the nervous system, we asked whether *hFUS*^+/+^; *Smn*^+/−^ mice might have an impairment in early motor functions. Accordingly, to assess motor functions and coordination starting from 2 days postnatal until 14 days of age, we performed a tube test, a righting reflex test and a negative geotaxis assay. As shown in Supplementary Figure 4, *hFUS*^+/+^ mice do not show any impairment of early motor functions compared to control mice, and the reduction of SMN levels does not affect these functions. Finally, no significant differences in the survival of animals are observed. Indeed, Kaplan-Mayer analysis of the cumulative survival of *hFUS*^+/+^; *Smn*^+/−^ mice perfectly matches that of *hFUS*^+/+^ mice, and animals with decreased SMN expression levels die at a similar age compared to the same animals with a wild-type amount of SMN (Fig. 5d and Supplementary Table 2).

### Molecular markers of *hFUS*^+/+^-related degeneration are not affected by SMN depletion

**Figure 6:**
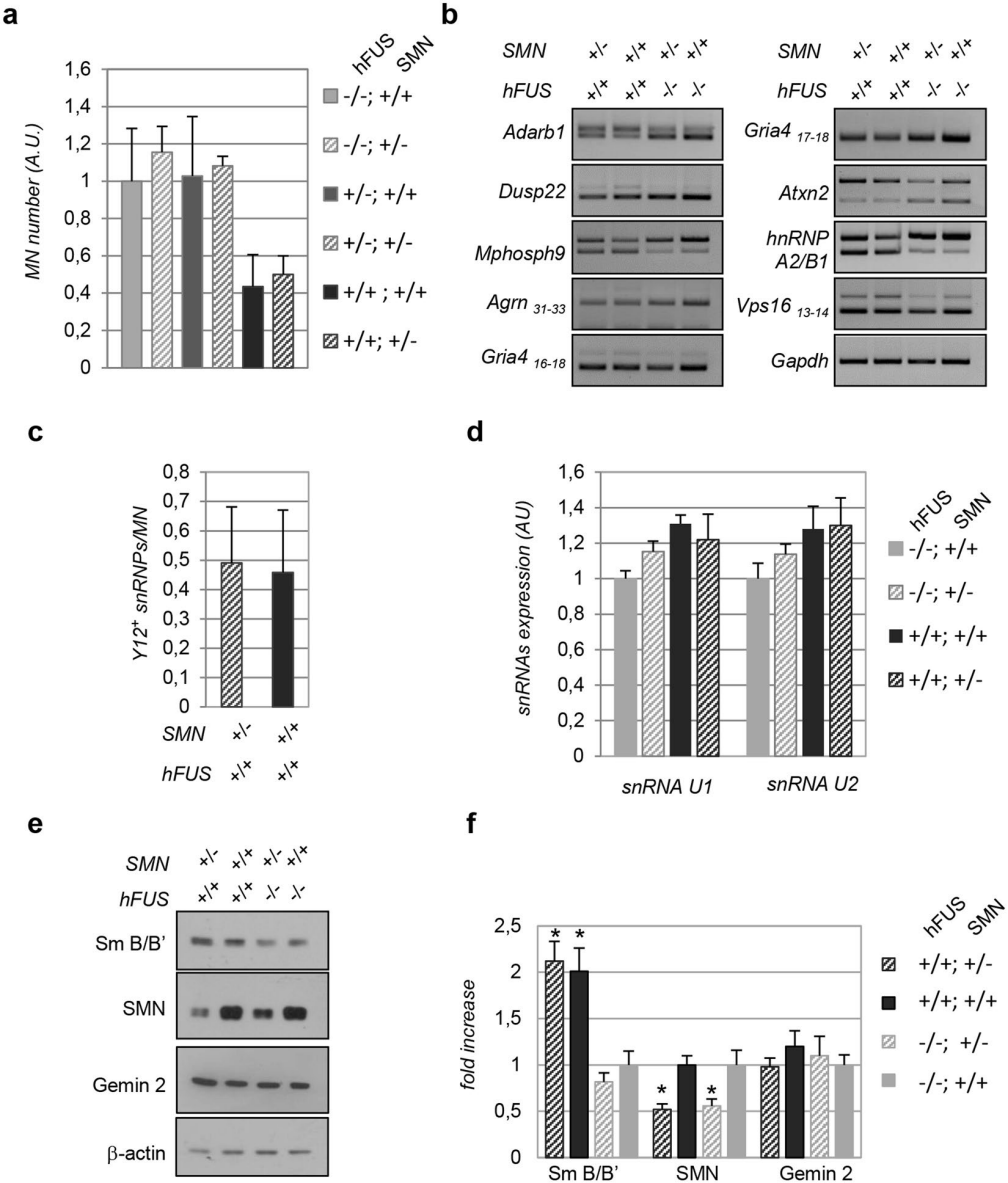
SMA molecular phenotypes are not affected by SMN depletion. (**a**) Quantitative analysis of Cresyl Violet stained motor neurons in the ventral horns of spinal cord from *hFUS*^+/+^; *Smn*^+/+^ and *hFUS*^+/+^; *Smn*^+/−^ mice at 40 days. n = 4 animals were used for each genotype. Values are reported as mean ± SD. (**b**) Alternative splicing patterns of the indicated target genes in endstage and control mice with the indicated genotypes. Results are representative of at least n = 3 independent experiments. Full-length agarose gels are presented in Supplementary Figure 9. (**c**) The number of Y12-positive snRNPs were counted in the motor neurons of *hFUS*^+/+^; *Smn*^+/+^ and *hFUS*^+/+^; *Smn*^+/−^ mice at 40 days. Results are reported as means ± SD. A total number of 50 motor neurons were scored. (**d**) RT-qPCR quantification of snRNA U1 and U2 in lysates from the spinal cords of the indicated mice. Means ± SE were normalized for the snRNA levels in control *hFUS*^−/−^ mice. (**e**) Expression levels of Gemin2, Sm B/B’, SMN, FUS and β-actin were assayed by Western blot on spinal cord lysates from 40 days-old mice of the indicated genotypes. The panel is representative of at least n = 3 independent experiments. Full-length blots are presented in Supplementary Figure 10. (**f**) The expression levels of SMN, Gemin2 and Sm B/B’ in *hFUS*^+/+^ mice were calculated by densitometric analysis of the bands from Western blots as in (**e**), normalised to β-actin levels and expressed as fold increases over the *hFUS*^−/−^; *Smn*^+/+^ control animals. Means ± SD are shown from n = 3 independent experiments. Values significantly different from relative controls are indicated with an asterisk when p ≤ 0.05.

According to the data reported above, SMN reduction does not affect the disease course of *hFUS*^+/+^ transgenic mice. To assess whether SMN hemizygosity does not influence the molecular events that characterize *hFUS*^+/+^ mice, we evaluated the animals for several phenotypes. Results shown in Fig. 6a indicate that SMN reduction does not impact on the total number of spinal cord motor neurons of *hFUS*^+/+^ transgenic mice. Indeed, at 40 days of age motor neurons are reduced to 60% in both *hFUS*^+/+^; *Smn*^+/−^ and *hFUS*^+/+^; *Smn*^+/+^ mice compared to spinal cords of control mice. This result, therefore, shows that a severe reduction of SMN levels is not sufficient to modify the degenerative process induced by human wild-type FUS overexpression. This conclusion is also supported by the analysis of alternative splicing events in *hFUS*^+/+^; *Smn*^+/−^ mice. As shown in Fig. 6b, the splicing of Dusp22, Mphosph9, Adarb1, hnRNP A2/B1, Gria4, Vps16, Atxn2 and Agrin, which are significantly affected in *hFUS*^+/+^ mice, is not further modified by SMN decrease. In line with these results, the number of nuclear snRNPs granules does not change in mice with different levels of SMN, as well as the expression pattern of the snRNAs and proteins that characterise transgenic *hFUS*^+/+^ mice are not changed by SMN reduction (Fig. 6c–f).

### SMN does not affect FUS-mediated neurodegeneration in *Drosophila* eye

**Figure 7:**
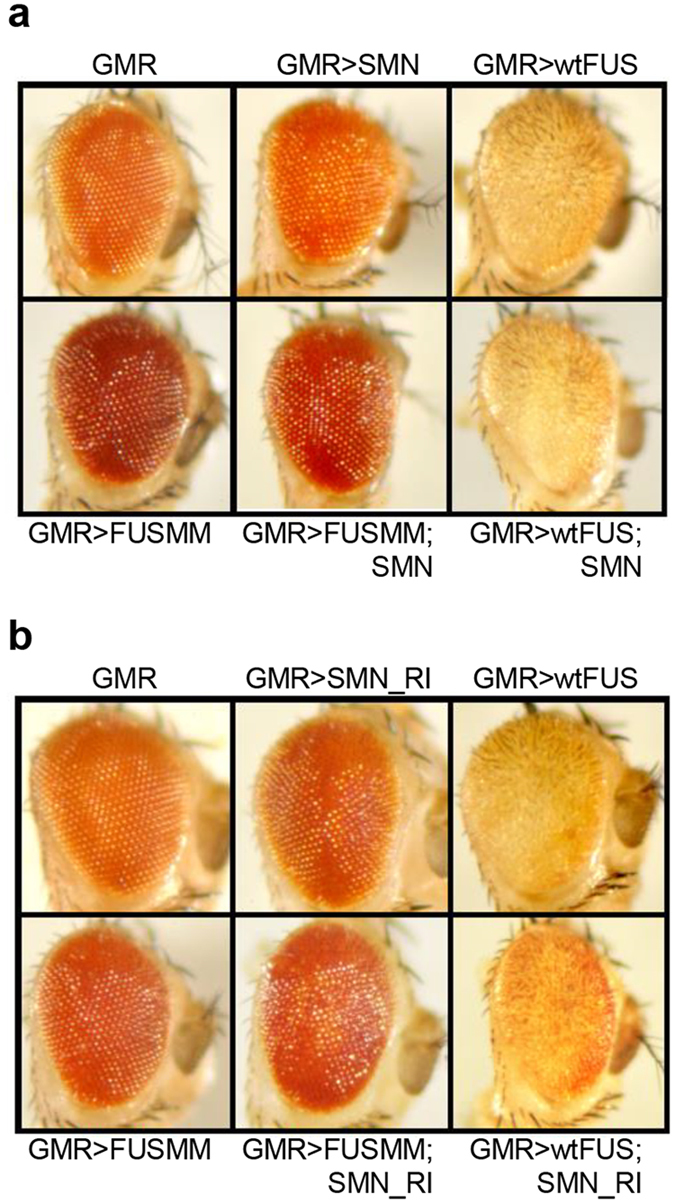
Alteration of SMN levels does not affect FUS-induced eye degeneration in Drosophila. (**a**) Representative pictures showing eyes from flies expressing the indicated human genes under the control of GMR GAL4. Fly lines carrying UAS::flag-FUS wild-type or UAS::flag-FUSMM transgenes on the second chromosome, together with GMR-GAL4, were grown at 29 °C. Flies expressing UAS::flag-SMN on its own were crossed with the GMR-GAL4 driver, at 29 °C. wtFUS induces a very strong eye degeneration, while expressing either FUSMM or SMN separately determines only very mild alterations of eye morphology. Significantly, the simultaneous expression of flag-SMN does not affect the strong eye degeneration caused by wtFUS. (**b**) Representative pictures showing eyes from flies expressing the indicated human genes under the control of GMR GAL4. RNAi mediated downregulation of *Smn* gene expression in fly eyes (SMN_RI) was obtained by expressing a specific RNAi construct (P (TRiP.HMC03832) attP40) at 29 °C, in a hemizygous background for *Smn* (Df (3 L) SmnX7/TM6B).

To further characterize the functional relationship between FUS and SMN, we moved to the *in vivo* model system of *Drosophila*, consisting in flies that co-express human FUS and SMN proteins under an UAS promoter. Utilizing the phiC31 integrase system, we have previously produced two different transgenic fly lines where the wild-type and a mutant form of FUS (FUSMM), which carries four independent ALS associated mutations (R521G, R522G, R524S, P525L), were inserted in the same genomic site, ensuring the same expression level. Thus, we generated recombinant Drosophilae where either wild-type or mutant FUS genes are located on the second chromosome together with the GAL4 driver GMR, to selectively express the transgenes in fly eyes. As already observed^25^, a strong and peculiar eye degeneration is detected in flies expressing wild-type FUS, while only minor alterations are visible in flies expressing FUSMM alone (Fig. 7a). Similarly, only a very mild degeneration is perceivable in eyes of flies over-expressing SMN. Importantly, when we combined the expression of wild-type FUS and SMN, which are both robustly expressed in the corresponding fly eyes (Supplementary Figure 5), no relevant modifications of the degeneration caused by FUS are detected. Additionally, no major effects are observed by the co-expression of SMN and FUSMM in fly eyes.

Finally, RNAi-mediated downregulation of dSMN in the eyes of hemizygous flies for *Smn*, by using GMR GAL4 driver (GMRGAL4; SmnRNAi/Df (3 L) SmnX7), modestly affects eye morphology (Fig. 7b). Similarly, the concurrent downregulation of SMN together with the expression of wtFUS does not induce any major effect on the eye degeneration. Again, no relevant effects are observed by the co-expression of SMN with FUSMM.

## Discussion

To address whether SMN function is implicated in a FUS model of ALS, in this work we investigated whether the ALS-like motor neuron pathology that characterizes mice overexpressing human wild-type FUS reproduces key molecular hallmarks that have been identified in SMA mice. Furthermore, we analysed whether and how SMN gene dosage impact on FUS-related disease phenotypes.

A number of conclusions can be drawn from the results reported above. Two independent observations sustain a clear overlap between SMA and FUS-mediated ALS. First, the number of assembled snRNPs that are present in the degenerating motor neurons in the lumbar spinal cords of *hFUS*^+/+^ mice is dramatically reduced, as it occurs in SMA mice, but also in other cellular and animal models of ALS^26,27^. While the pathological significance of such a reduction is far from being clear, in *hFUS*^+/+^ mice it is evident that this effect is neither due to an improper expression of the major constituents of the snRNPs, nor to their improper assembly. Indeed, no major differences in the steady state levels of snRNAs, or in their binding to Sm proteins were observed. This is different to what has emerged from studies on SMA mice, where a reduction in the levels of U snRNAs, as well as in their assembling into snRNPs, accounts for the alterations in nuclear Gems observed^12^. Thus, it is conceivable that snRNP reduction in FUS mice is the consequence of a different mechanism. A defect in the proper localization of snRNPs, as it occurs in cellular systems mimicking FUS-dependent ALS, as well as in fibroblasts from patients^8,10^, represents a plausible option. Since defects in the shuttling of proteins and RNAs between the nucleus and the cytoplasm are emerging as a crucial issue in ALS pathogenesis^28^, we suggest that the proper regulation of nucleocytosolic shuttling of snRNAs and snRNPs may be also affected by wild-type FUS overexpression, similarly to what has been observed in presence of FUS mutant proteins.

A second important similarity between the pathogenic mechanisms accounting for SMA and FUS-ALS is related to alterations in mRNA splicing. Indeed, changes in the alternative splicing of selected pre-mRNAs, that encode proteins involved in the maturation, stability and function of neuromuscular junctions, are believed to underlie SMA pathogenesis. Out of 20 SMA-linked alternative spliced mRNAs monitored, 10 are clearly affected also in diseased FUS mice (Supplementary Table 1). This strongly argues for a shared pathogenic mechanism between FUS-ALS and SMA, and the defects in snRNP distribution might explain this result. Importantly, the bulk of alternative splicing alterations observed in *hFUS*^+/+^ mice does not significantly occur in G93A-SOD1 mice, nor in mice modelling SBMA, which are both characterized by the degeneration of motor neurons in the spinal cord. These results strongly support the conclusion that the observed alterations might have a primary and causative role in the spinal cord pathology characterizing hFUS transgenic mice, similarly to what has been proposed for SMA mice.

Among the genes whose pre-mRNAs are characterised by modified alternative splicing, some deserve particular attention. ADAR2 (adenosine deaminase acting on RNA 2), an enzyme responsible for RNA editing through site-specific deamination of adenosines to inosine and that has a role in the editing of the mRNA that encodes for the GluA2 subunit of the (AMPA) glutamate receptor, is profoundly altered in its splicing. Even though further analyses are needed to unveil the role of splicing variations on ADAR2 function, this finding is to be considered particularly interesting as ADAR2 deficiency occurs in the majority of ALS cases, including an ALS patient with a FUS (P525L) mutation^29,30^. Similarly, hnRNP A2/B1, that has been indicated as a possible major player in some familial cases of ALS^31^ and that is impressively affected in *hFUS*^+/+^ mice, deserves further investigation.

Overall, these results strongly support a role of SMN in ALS pathogenesis due to overexpression of wild-type FUS. Yet, how FUS and SMN functionally interact to cause ALS disease is not clear. In order to elucidate this issue, we therefore investigated whether SMN expression might affect the pathological phenotypes that characterize disease onset and progression in *hFUS*^+/+^ mice. In particular, since the shortage of SMN is the actual cause of motor neuron degeneration in SMA, we analysed whether a reduction of SMN could produce any effect on asymptomatic heterozygous hFUS mice, or modify the phenotypes in diseased hFUS homozygous animals. Surprisingly, we did not observe any of these effects. Motor performance, as well as survival of *hFUS*^+/−^ mice, are not affected by SMN reduction, and these mice are undistinguishable from control mice. Similarly, disease progression of *hFUS*^+/+^; *Smn*^+/−^ mice, as measured by weight, grip test and cumulative survival analysis, completely overlaps that of *hFUS*^+/+^ mice.

The finding that a 50% reduction in the amount of SMN is not sufficient to modify disease progression in mice might imply that a further reduction in SMN might be needed to influence the disease course. However, a consistent reduction of dSMN in flies overexpressing wild-type FUS is not sufficient to modify the degeneration observed in *Drosophila* eyes. Most interestingly, a relevant expression of exogenous SMN does not rescue the neurodegeneration caused by the expression of wild-type FUS in *Drosophila* eyes. Thus, other possibilities should be taken into account. In particular, the observation that the approach used in this work is not efficient in affecting SMN-regulated pathways, including the alternative splicing of genes that are common to FUS-ALS and SMA models and that therefore appear as potential candidates in motor neuron degeneration in both diseases, suggests that the splicing regulation exerted by FUS insists on the same molecular pathway as SMN, and that FUS may act downstream of SMN function and independently from SMN-assisted regulation of snRNP biosynthesis and assembly. This conclusion is further supported by observations derived from cultured cells and patient-derived fibroblasts^8,10,11^, where a defect in the proper localization of snRNPs, rather than an impairment in their assembly, suggests a role for FUS in snRNP nucleocytosolic trafficking. Intriguingly, SMN reduction in the G93A-SOD1 mouse model of ALS is effective in modifying the phenotypic severity in these ALS mice^26^, and the overexpression of SMN in SOD1 mice, as well as in mice transgenic for an ALS –associated mutant TDP-43, attenuates motor neuron degeneration^27,32^. Overall, these data clearly support the idea that ALS pathogenesis might involve a dys-regulation of spliceosomal snRNP functions, but they also suggest that SOD1 and TDP-43 might target these functions differently from FUS.

Yet, other possibilities should be also considered. Indeed, recent data showed that FUS acts as a classical hnRNP, thereby promoting or inhibiting splicing in relation to its positioning in the intronic/exonic regions, and that FUS deletion or mutations linked to ALS interfere with this process, affecting the splicing pattern of target genes^11^. On these grounds, it is possible that FUS and SMN regulate the alternative splicing of common targets by independent pathways that converge on the same set of genes, that are relevant for motor neurons, by mechanisms that still need to be identified. Whether these mechanisms involve the ‘minor’ spliceosome, that catalyses the removal of U12-type spliceosomal introns, as a common, preferential target, which is suggested by a number of studies on SMA mice^15–17^ as well as from recent data on ALS-linked mutant FUS^11^, is still an open question.

In summary, we have shown here that the pathogenesis of FUS-associated ALS recapitulates *in vivo* crucial molecular features of the degenerative process that characterise mouse models of SMA. Among these features, the alterations in the splicing pattern of a number of genes that have important roles in motor neuron function, as a possible consequence of defects in snRNP trafficking, suggest that these genes might be involved in the selective degeneration of motor neurons that is common to both diseases. Yet, alterations in SMN expression do not modify the disease course nor the molecular phenotypes analysed. While these data provide a further support to the notion that FUS and SMN act on the same pathway, they also indicate the existence of a complex interplay between FUS and SMN in the regulation of alternative splicing and that strategies targeting this pathway deserve further investigations.

## Methods

### Animal models

Adult Tg (Prnp-FUS) WT3Cshw/J mice expressing hemagglutinin-tagged human wild-type FUS^21^ and adult B6.129P2 (Cg)-Smn1<tm1Msd>/J, expressing hemizygous deletion of SMN1 protein^33^, were obtained from Jackson Laboratories. Animals were housed in our indoor animal facility at constant temperature (22 ± 1 °C) and relative humidity (50%) with 12-h light cycle (light 7 am–7 pm). Both mouse lines were maintained in hemizygousity on the same C57BL/6 genetic background. Hemizygous FUS mice were backcrossed to obtain homozygous mice, used as experimental subjects.

To obtain FUS mice with reduced levels of SMN, heterozygous hFUS^+/−^ mice were crossed with Smn^+/−^ mice. The resulting FUS^+/−^; Smn^+/−^ mice were further crossed with hFUS^+/−^ mice to finally produce hFUS^+/−^; Smn^+/−^ animals. Food and water were freely available. When animals showed symptoms of paralysis, wet food was given daily into the cages for easy access to nutrition and hydration. Mice were genotyped by PCR analysis of tissue extracts from tail tips. Hemizygous FUS mice were identified using PCR primers: Fwr5′-AGGGCTATTCCCAGCAGAG-3′, Rev5′-TGCTGCTGTTGTACTGGTTCT-3′. Homozygous FUS mice were genotyped by qPCR using following primers: Fwr5′-GCCAGAACACAGGCTATGGAA-3′ and Rev5′-GTAAGACGATTGGGAGCTCTG-5′. SMN mice were screened by PCR using primers For5′-TTTTCTCCCTCTTCAGAGTGAT-3′, Rev5′-CTGTTTCAAGGGAGTTGTGGC-3′ and RevMut5′-AACGCCAGGGTTTTCC-3′. SOD1-G93A and SBMA mice were previously described^23,34^. All animal procedures were performed according to the European Guidelines for the use of animals in research (2010/63/EU) and the requirements of Italian laws (D.L. 26/2014). The ethical procedure was approved by the Italian Ministry of Health (protocol number 92/2014 PR/G). All efforts were made to minimize animal suffering and the number of animals necessary to produce reliable results.

Transgenic flies expressing Flag-FUSWT, Flag-FUSMM and Flag-SMN have been already described^25,35^. RNAi fly line targeting SMN gene expression (y1sc*v1; P (TRiP.HMC03832) attP40) was obtained from Bloomington stock center as well as Gal4 driver utilized (http://flybase.bio.indiana.edu/). To achieve the strongest possible RNAi mediated downregulation of smn gene expression in fly eye, we utilized a fly line that expresses the RNAi construct in a genetic background with halved genetic content of *Smn* (P (TRiP.HMC03832) attP40; Df (3 L) SmnX7/TM6B). Drosophila stocks and crosses were maintained on standard Drosophila medium at 29 °C. The following genotypes were utilized: wtFUS (UAS::Flag-wtFUS, GMR-Gal4; TM6B/MKRS); FUSMM (UAS::Flag-FUSMM, GMR-Gal4; TM6B/MKRS); SMN_RI (P (TRiP.HMC03832) attP40; Df (3 L) SmnX7/TM6B); SMN (UAS::Flag-SMN; TM6B/MKRS); GMR-Gal4 (GMR-Gal4/CyO; TM6B/MKRS).

### Histology and indirect immunofluorescence

Diseased end-stage mice (P40) and age-matched controls were anaesthetized by intraperitoneal injection of Rompum (xylazine, 20 mg/ml, 0.5 ml/kg Bayer, Milan, Italy) and Zoletil (tiletamine and zolazepam, 100 mg/ml, 0.5 ml/kg; Virbac, Milan, Italy) and transcardially perfused with saline solution followed by 4% paraformaldehyde (PFA) in phosphate buffer (PB; 0.1 M; pH 7.4). Spinal cords were immediately dissected and post-fixed in 4% PFA for 4 hours, incubated in 30% sucrose in PB solution for 12 hours at 4 °C and cut into 30 µm thick slices with a freezing microtome. For motor neuron count, Nissl staining was performed on lumbar spinal cord (L3–L5) of 4 mice per genotype. After staining with 1% Cresyl violet and gradual dehydration in 50–100% alcohol, slices were cleared in xylene and coverslipped with Eukitt (Sigma-Aldrich). To compare motor neuron number, one slice every 3rd, for a total of 10 sections, was analyzed with Stereo Investigator System (MicroBrightField Europe e.K., Magdeburg, Germany) and polygonal-shaped neurons, bigger than 200 µm^2^, with well-defined cytoplasm, nucleus and nucleolus were counted. For immunohistochemistry, lumbar spinal cord slices from at least three animals per genotype were incubated 24 hours at 4 °C with primary antibodies diluted in PB, 0.3% Triton X-100 and then for 2 hours at room temperature with appropriate secondary antibody, diluted in the same solution. After three rinses, 5 min each, in PB, nuclei were stained with 1 µg/ml Höechst 33258 (Sigma-Aldrich) for 5 minutes. For snRNPs localization, a 72 hours incubation with primary Y12 antibody was employed in the same conditions. For the staining of neuroinflammation markers, an hybridization step of one hour in 10% normal donkey serum (NDS) in PBS, 0.3% Triton X-100 (PBS-TX) was performed before the incubation with primary antibodies diluted in 2% NDS in PBS-TX for 24 hours at 4 °C. Appropriate secondary antibodies were used for three hours in 2% NDS in PBS-TX. Antibodies used: Rabbit anti-FUS (Bethyl), Goat anti-ChAT (Millipore), Mouse Y12 (Novus Biological), Rabbit anti-ADAR2 (Proteintech), Rabbit anti-Iba-1 (Wako), Mouse anti-GFAP (Cell Signaling), Alexa Fluor 488 goat anti-mouse (Invitrogen), Alexa Fluor Cy3 goat anti-rabbit (Jackson ImmunoResearch Laboratories) and Alexa Fluor 568 donkey anti-goat (Invitrogen). Samples were analysed with a Zeiss LSM 510 Confocal Laser Scanning Microscope equipped with 40x and 63x objectives and images were processed using ZEN 2009 (Carl Zeiss) and Adobe Photoshop software.

### Assessment of motor functions and health

Starting from the first week of age, *hFUS*^+/+^; *Smn*^+/+^, *hFUS*^+/+^; *Smn*^+/−^ and control mice were assessed for weight, general health and survival on a weekly basis. Animals that failed to splay hind limbs normally when lifted by their tail were monitored daily and euthanized as end-stage mice when they could not obtain food or water by themselves. For evaluation of early motor functions, newborn mice, for a total of 6 per genotype, were subjected to tube test, negative geotaxis and righting reflex test on a daily basis starting from 2 days of age until 14 days of life. A detailed description of these tests is found in ref.^36^. Every testing day, at the same hour, neonates and dams were brought to the experimental room and left undisturbed for 10 minutes. Pups were tested one at a time and placed in a warm box until the end of the session, when they were mixed with the cage bedding before the returning to the cage to avoid maternal rejection. To assess motor functions on mice older than 14 days of life, inverted grid test and Rotarod test were performed as described^34^.

### Quantification of U snRNAs

For the analysis of U snRNAs bound to Sm core, at least six animals per genotype were euthanized at P40 and spinal cords were lysed in Lysis Buffer (20 mM NaCl pH 8.0, 250 mM NaCl, 1.5 mM MgCl2, 0.2% Triton X-100) containing 0.2 U/µl RNAsin (Pomega) and protease inhibitors cocktail (Sigma-Aldrich). Equal amounts of protein extracts from *hFUS*^+/+^ mice and controls were incubated at 4 °C for 2 hours with protein G-agarose beads (Roche) conjugated primary antibody Y12. Mouse IgGs (Santa-Cruz) were used as negative control. After 3 washes with 20 mM Tris HCl pH 8.0, 50 mM NaCl and 1.5 mM MgCl_2_, immunocomplexes were resuspended in RNA Elution Buffer (0.2 M NaOAc pH 5.0, 0.2% SDS, 1 mM EDTA) and *in vitro*-transcribed BC200 RNA was added as internal efficiency control. Bound RNAs, as well as total RNAs from cell lysates, were PCA-extracted and reverse transcribed with Super Script III First Strand kit (Invitrogen) using specific primers for U snRNAs. qPCR reactions were performed with the Light Cycler 480 SYBR Green System (Roche) using BC-1 RNA as unrelated negative control. Cp values were calculated using the ‘second derivative max’ algorithm of the Lightcycler software. SnRNA quantities were calculated from these Cps using experimentally determined amplification efficiencies, and then normalized for the BC200 control and for the amount of snRNA from cell lysates used for the respective immunoprecipitation. The quantification of total U snRNAs from spinal cords of end-stage diseased mice and controls (n = 6 per genotype) was performed by extracting total RNAs with Trizol Reagent (Invitrogen), followed by DNAse treatment (Promega), PCA-extraction and retrotranscription with specific primers for U snRNAs (Super Script III First Strand kit, Invitrogen). BC-1, Hsp70 and β-actin RNAs were initially used as housekeeping genes. As they gave consistent results, BC-1 was used for most of the analysis. The list of oligonucleotides used is provided as Supplementary Table 3.

### Immunoblotting

Spinal cords of at least 5 animals per genotype were dissected and lysed in RIPA buffer (50 mM Tris HCl pH 7.4, 250 mM NaCl, 1 mM EDTA, 5 mM MgCl2, 1% Triton X-100, 0.25% Na-Deoxycholate, 0.1% SDS, protease inhibitor cocktail). After 2 × 10″ sonication cycles, samples were incubated on ice and then centrifuged at 18000 × g for 20′ at 4 °C. Supernatants were then quantified with Bradford protein assay (Bio-rad) and resuspended in Laemmli Buffer before SDS-PAGE (Sigma-Aldricht). Antibodies used: rabbit anti-FUS (Bethyl), mouse anti-GFAP (Cell Signaling), mouse anti-Iba1 (Wako), rabbit anti-Gemin2 (Proteintech), mouse anti-Sm B/B’ (Thermo Scientific), mouse anti-SMN (BD), mouse anti-β-actin (Sigma).Anti‐rabbit and anti‐mouse IgG peroxidase‐conjugated secondary antibodies were from Bio Rad.

### Alternative splicing analysis

Total spinal cords RNAs from 5 mice per genotype were extracted with Trizol reagent, PCA-precipitated and retrotranscribed with Im-Prom II reverse transcription system (Promega). PCR reactions were performed using Biomix Red (Bioline) and oligonucleotides listed in Supplementary Table 4. PCR products were run in 2% agarose gels and visualized by ethidium bromide staining. Images were acquired on a Geldoc imaging system (Biorad), and bands were quantified using the ImageJ software (National Institute of Health).

### Statistical analysis

Statistical analysis was performed with an unpaired two‐tailed Student’s t‐test. Values significantly different from the relative control are indicated with asterisks. P‐values ≤ 0.05 or 0.01 were considered significant. Mice survival was analysed with the Kaplan-Meier Graph followed by log-rank statistics, using MedCalc Statistical Software version 15.8.

## Electronic supplementary material

Supplementary Material
