# Publisher Correction: Functional interaction between FUS and SMN underlies SMA-like splicing changes in wild-type hFUS mice

**DOI:** 10.1038/s41598-018-25176-3

**Published:** 2018-04-30

**Authors:** Alessia Mirra, Simona Rossi, Silvia Scaricamazza, Michela Di Salvio, Illari Salvatori, Cristiana Valle, Paola Rusmini, Angelo Poletti, Gianluca Cestra, Maria Teresa Carrì, Mauro Cozzolino

**Affiliations:** 1 Fondazione Santa Lucia IRCCS, 00143 Rome, Italy; 2 Dipartimento di Biologia, Università di Roma “Tor Vergata”, Rome, Italy; 3 Istituto di Farmacologia Traslazionale (IFT), CNR, 00133 Rome, Italy; 4 Istituto di Biologia e Patologia Molecolari (IBPM), CNR, 00185 Rome, Italy; 5 Dipartimento di Biologia e Biotecnologia “Charles Darwin”, Università di Roma “Sapienza”, 00185 Rome, Italy; 6 Istituto di Biologia Cellulare e Neurobiologia (IBCN), CNR, 00143 Rome, Italy; 7 Dipartimento di Scienze Farmacologiche e Biomolecolari (DiSFeB), Centro di Eccellenza sulle Malattie Neurodegenerative, Università degli Studi di Milano, 20133 Milan, Italy

Correction to: *Scientific Reports* 10.1038/s41598-017-02195-0, published online 17 May 2017

The original version of this Article contained errors in the name of the author Mauro Cozzolino, which was incorrectly given as Maur o Cozzolino.

These errors have now been corrected in the PDF and HTML versions of the Article.

