# Supplementary material for: Functional interaction between FUS and SMN underlies SMA-like splicing changes in wild-type hFUS mice
Source: Sci Rep. 2017 May 17;7:2033. doi: 10.1038/s41598-017-02195-0 (PMC5435706; doi:10.1038/s41598-017-02195-0)

Functional interaction between FUS and SMN underlies SMA-like splicing changes in wild-type hFUS mice

Alessia Mirra [a,b], Simona Rossi [c], Silvia Scaricamazza [a,b], Michela Di Salvio [d,e], Illari Salvatori [a], Cristiana Valle [a,f], Paola Rusmini [g], Angelo Poletti [g], Gianluca Cestra [d,e], Maria Teresa Carrì [a,b,*], Mauro Cozzolino [c,*,#]

a Fondazione Santa Lucia IRCCS, 00143 Rome, Italy,

b Dipartimento di Biologia, Università di Roma “Tor Vergata”, Rome, Italy,

c Istituto di Farmacologia Traslazionale (IFT), CNR, 00133 Rome, Italy,

d Istituto di Biologia e Patologia Molecolari (IBPM), CNR, 00185 Rome, Italy

e Dipartimento di Biologia e Biotecnologia "Charles Darwin", Università di Roma "Sapienza", 00185 Rome, Italy

f Istituto di Biologia Cellulare e Neurobiologia (IBCN), CNR, 00143 Rome, Italy

g Dipartimento di Scienze Farmacologiche e Biomolecolari (DiSFeB), Centro di Eccellenza sulle Malattie Neurodegenerative, Università degli Studi di Milano, 20133 Milan, Italy

*These authors should be considered joint senior authors

[#]To whom correspondence should be addressed: Mauro Cozzolino, Institute of Translational Pharmacology, National Research Council (CNR), Via del Fosso del Cavaliere 100, 00133, Rome, Italy. Tel: +39-06-49934418; Fax: 49+39-06-49934257; Email: mauro.cozzolino@ift.cnr.it

SUPPLEMENTARY MATERIALS

Supplementary Figure 1. Overexpression of wild-type human FUS causes ALS-like disease and early lethality in mice.

a. Western blot to detect FUS on spinal cords from 40 days homozygous (+/+), heterozygous (+/-) and non transgenic (-/-) mice overexpressing human wild-type FUS (hFUS). mFUS: endogenous FUS. The expression levels FUS (endogenous plus exogenous) in the indicated mice were calculated by densitometric analysis of the bands from Western blots as in (a), normalised to β-actin levels and expressed as fold increases over the $hFUS^{-/-}$ control animals. Means ± SD are shown from n=3 independent experiments. Values significantly different from relative controls are indicated with an asterisk when $p \leq 0.05$. Full-length blots are presented in Supplementary Figure 11.

b. Control (-/-) mice, as well as homozygous (+/+) and heterozygous (+/-) hFUS animals were weighted once a week starting from 3 weeks of age. n=14 animals for each genotype were scored. **c.** A grip test was used to assay the motor abilities of $hFUS^{-/-}$, $hFUS^{+/-}$ and $hFUS^{+/+}$ mice, starting from 20 days of age. Results from n=6 animals were scored. **d.** Kaplan-Meier analysis was used to assess the survival probability of the indicated mice. Log–rank test was used to compare $hFUS^{-/-}$, $hFUS^{+/-}$ and $hFUS^{+/+}$ animals. Equal numbers of males and females were analysed for each genotype. No sex-dependence was observed and therefore data were pooled. **e.** Astrocytes (GFAP) and microglia (iba-1) activation was detected in lumbar spinal cords slices from control (-/-) and $hFUS^{+/+}$ mice at 40 days of age. Magnifications of selected areas are shown in the insets. Scale bar: 100 µm. **f.** Lysates from 40 days old mice with the indicated genotypes were analyzed in Western blot with the indicated antibodies. The expression levels of GFAP and iba1 in the indicated mice were calculated by densitometric analysis of the bands from Western blots, normalised to β-actin levels and expressed as fold increases over the $hFUS^{-/-}$ control animals. Means ± SD are shown from n=3 independent experiments. Values significantly different from relative controls are indicated with an asterisk when $p \leq 0.05$. Full-length blots are presented in Supplementary Figure 11

g. Spinal cord sections from the lumbar spinal cords of *hFUS*$^{-/-}$ and *hFUS*$^{+/+}$ mice (40 days) were stained with Cresyl Violet to assess motor neuron numbers. Representative sections are shown for each genotype. **h.** Quantitative analysis of motor neurons in the ventral horns of spinal cord from the indicated mice. n=4 animals were used for each genotype. Values are reported as mean ± SD. Values significantly different from relative controls are indicated with an asterisk when $P \leq 0.05$.

Supplementary Figure 2. Overexpressed hFUS does not form cytoplasmic inclusions.

Lumbar spinal cord sections from end-stage transgenic *FUS*$^{+/+}$ animals were immunostained with antibodies anti-ChAT, FUS and Sm. Nuclei were stained with DAPI. A magnification of the selected areas is shown on the right. Scale bar: 20 μm

***Supplementary Figure 3*. *Analysis of SMN levels in hFUS*$^{+/+}$*; Smn*$^{+/+}$ and *hFUS*$^{+/+}$*;Smn*$^{+/-}$ mice.**

a. Western blot analysis using antibodies against FUS and SMN. β-actin was analysed as loading control. Spinal cords lysates from end-stage *hFUS*$^{+/+}$*;Smn*$^{+/+}$ *and hFUS*$^{+/+}$*;Smn*$^{+/-}$ were used. Non transgenic mice (*hFUS*$^{-/-}$*; SMN*$^{+/+}$) and heterozygous mice (*hFUS*$^{-/-}$*; SMN*$^{+/-}$) were employed as controls. hFUS: exogenous human FUS. mFUS: endogenous mouse FUS. Full-length blots are presented in Supplementary Figure 12**. b.** The expression levels of SMN and FUS (endogenous plus exogenous) in the indicated mice were calculated by densitometric analysis of the bands from Western blots as in (a), normalised to β-actin levels and expressed as fold increases over the *hFUS*$^{-/-}$;*Smn*$^{+/+}$ control animals. Means ± SD are shown from n=3 independent experiments. Values significantly different from relative controls are indicated with an asterisk when $p \leq 0.05$.

Supplementary figure 4. Evaluation of early motor phenotypes in hFUS*$^{+/+}$*; Smn*$^{+/+}$ *and hFUS*$^{+/+}$*;Smn*$^{+/-}$ *mice.

A battery of three tests was used to evaluate early motor symptoms. All the tests were performed starting from 2 days of life until 14, every other day, in the same room and at the same hour. *hFUS*$^{+/+}$;

Smn$^{+/-}$ and *hFUS*$^{+/+}$; *Smn*$^{+/+}$ mice were tested and compared to non transgenic animals (NT). Pups were separated from the mothers immediately before the test and kept warm during the test. **a.** Tube test. **b.** Righting reflex test. **c.** Negative geotaxis test. At least n=6 animals for each genotype were used. Results are presented as mean ± SD.

Supplementary Figure 5. Expression levels of wtFUS, FUSMM and SMN human proteins in fly eyes.

Heads from flies expressing the indicated transgenes under GMR were isolated and homogenized. Protein extracts were separated on SDS-PAGE and the presence of each transgene was evaluated by western blotting utilizing anti-flag antibodies. β-actin was used as a loading control. Full-length blots are presented in Supplementary Figure 13

Supplementary Figure 6. Original Western blots for Figure 1b.

Full length Western blots of spinal cord lysates from *hFUS*$^{+/+}$ and *hFUS*$^{-/-}$ mice presented in Figure 1b. Rectangles refer to the regions of the digitalized films that were used to generate figure panels. Scanned films were processed using Adobe Photoshop CC and Office Power Point.

Supplementary Figure 7. Original agarose gels for Figure 3a.

Full length agarose gels of PCR products of spinal cord lysates from *hFUS*$^{+/+}$ and *hFUS*$^{-/-}$ mice presented in Figure 1b. Rectangles refer to the regions of the original images that were used to generate figure panels. Ethidium bromide stained gels were acquired using a Geldoc from Biorad, and digitalized images were processed using Adobe Photoshop CC and Office Power Point. MK: Marker VI, Roche.

Supplementary Figure 8. Original agarose gels for Figure 4a,c.

Full length agarose gels of PCR products of spinal cord lysates from SOD1 G93A and SBMA mice presented in Figure 4a,c. Rectangles refer to the regions of the digitalized films that were used to generate figure panels. Ethidium bromide stained gels were acquired using a Geldoc from Biorad, and digitalized images were processed using Adobe Photoshop CC and Office Power Point.

Supplementary Figure 9. Original agarose gels for Figure 6b.

Full length agarose gels of PCR products of spinal cord lysates from the indicated mice presented in Figure 6b. Rectangles refer to the regions of the original images that were used to figure panels. Ethidium bromide stained gels were acquired using a geldoc from Biorad, and digitalized images were processed using Adobe Photoshop CC and Office Power Point.

Supplementary Figure 10. Original Western blots for Figure 6e.

Full length Western blots of spinal cord lysates from the indicated mice presented in Figure 6e. Rectangles refer to the regions of the digitalized films that were used to generate figure panels. Scanned films were processed using Adobe Photoshop CC and Office Power Point.

Supplementary Figure 11. Original Western blots for Supplementary Figure 1a and 1f.

Full length Western blots of spinal cord lysates from the indicated mice presented in Supplementary Figure 1a and 1f. Rectangles refer to the regions of the digitalized films that were used to generate figure panels. Scanned films were processed using Adobe Photoshop CC and Office Power Point.

Supplementary Figure 12. Original Western blots for Supplementary Figure 3a.

Full length Western blots of spinal cord lysates from the indicated mice presented in Supplementary Figure 3a. Rectangles refer to the regions of the digitalized films that were used to generate figure panels. Scanned films were processed using Adobe Photoshop CC and Office Power Point.

Supplementary Figure 13. Original Western blots for Supplementary Figure 5.

Full length Western blots of lysates from eyes from the indicated flies presented in Supplementary Figure 5. Images were acquired with a Chemidoc (Roche). Rectangles refer to the regions of the acquired images that were used to generate figure panels. Acquired images were processed using Adobe Photoshop CC and Office Power Point.

SUPPLEMENTARY MATERIALS

Supplementary Table 1

Summary of the alternative splicing events analysed. The presence or absence of significant variations in the alternative splicing pattern are indicated by "+" or "-", respectively. na: not addressed. Numbers in superscript refers to papers where alternative splicing changes in SMA have been reported for the indicated genes.

(1). Zhang et al., 2013. PNAS. 110:19348-19353.

(2). Lotti et al., 2012. Cell. 151:440-454.

(3). Boulisfane et al., 2011. Hum Mol Genet. 20:641-648.

Gene name	*SMA*	*ALS wtFUS*	*ALS G93A SOD1*	*SBMA AR113Q*
Adarb1	+ (1)	+	-	-
Camk2d	+ (1)	-	-	-
Dusp22	+ (1)	+	-	-
Mphosph9	+ (1)	+	-	-
Agrin $_{31\text{-}33}$	+ (1)	+	-	-
Agrin $_{31\text{-}34}$	+ (1)	+	+	-
Gria4 $_{16\text{-}18}$	+ (1)	+	-	-
Gria4 $_{17\text{-}18}$	+ (1)	+	+	-
Atxn2	+ (1)	+	+	-
Uspl1	+ (1)	-	*na*	*na*
Mark2	+ (1)	-	*na*	*na*
Vps16 $_{9\text{-}10}$	+ (2)	-	*na*	*na*
Vps16 $_{13\text{-}14}$	+ (2)	-	-	-
C19orf54	+ (2)	+	-	-
Parp1	+ (2)	+	*na*	*na*
Clcn7	+ (2)	+	*na*	*na*
Harsl	+ (2)	-	*na*	*na*
Tmem41b	+ (2)	-	*na*	*na*
Atxn10	+ (3)	-	*na*	*na*
Mapk8	+ (3)	-	-	-
Thoc2	+ (3)	-	*na*	*na*
hnRNP A2/B1	*na*	+	-	-
hnRNP D	*na*	-	*na*	*na*

Supplementary Table 2

Statistical analysis of the survival probability curves shown in Figure 5

genotype (hFUS;Smn)	***-/-;+/-***	***-/-;+/+***	***+/-;+/-***	***+/-;+/+***	***+/+;+/-***	***+/+;+/+***
number of animals	32	58	32	36	17	71
median survival	-	-	-	-	41	40
mean survival ± SD	-	-	-	-	40,6±5,3	40,2±5,8
log rank *+/+;+/+ vs -/-;+/+*	Chi-squared	139,9046				
	DF	1				
	Significance	P < 0,0001				
log rank *+/+;+/+ vs +/+;+/-*	Chi-squared	0,1313				
	DF	1				
	Significance	P = 0,7171				

Supplementary Table 3
Oligonucleotides for qPCR analysis of snRNAUs abundance

Name	Forward Sequence (5'→3')	Reverse Sequence (5' →3')
HSP-70	GTGTGAGAGGGCCAAGAGG	ATGCCCTCGAACAGAGAGTC
ACTIN	CTAAGGCCAACCGTGAAAAG	ACCAGAGGCATACAGGGACA
BC-1	CTGGGTTCGGTCCTCAG	TGTGTGTGCCAGTTACCT
BC-200	CCTGCCTGGGCAATATAGC	GCTTTGAGGGAAGTTACGC
U1	GATACCATGATCACGAAGGTGGT	CACAAATTATGCAGTCGAGTTTC
U2	TTTGGCTAAGATCAAGTGTAGTATCTGTT	AATCCATTTAATATATTGTCCTCGGATAG
U11	GTGCGGAATCGACATCAAG	GGACCAACGATCACCAG
U12	AATAACGATTCGGGGTGACG	GCAACCTATACCCGCTCAAA
U4	GCGCGATTATTGCTAATTGAA	AAAAATTGCCAATGCCGACT
U5	GGTTTCTCTTCAGATCGTATAAAT	CTCAAAAAATTGGTTTAAGACTCAG
U6	GCTTCGGCAGCACATATACTAAAA	ACGAATTTGCGTGTCATCCT
U4atac	GCGCATAGTGAGGGCAGTACT	GCACCAAAATAAAGCAAAAGCTCTA
U6atac	AGGTTAGCACTCCCCTTGACAA	TGGCAATGCCTTAACCGTATG

Supplementary Table 4
Oligonucleotides used in Alternative Splicing Analysis. For each gene, the exons where the primers anneal are indicate

Gene name	Forward Sequence (5'→3')	Reverse Sequence (5' →3')
Adarb1(E7-E8)	GGTTCCGGCTGAAGGA	CCTCGCCAGACTCTATTTTC
Camk2d (E20-E22)	GCGGGATGCCAAAGAC	ATTACACGTAGAACCTTCACAAC
Gria4 (E16-E18)	CGGGGGAGGTGACTC	CTTCATTCTCTTCGCCTCTG
Gria4 (E17-E18)	CCCAAGGACTCGGGAAG	CTTCATTCTCTTCGCCTCTG
Ataxin 2 (E9-E11)	TCAAGAGCTGCTTCTCACA	AGGAGCAGCTGCTTCAC
C19orf54 (E4-E6)	ACACCGCACAGGGAGAAATGTT	CAGTGGGCCTTATGGCCTTTCTTT
Vps 16 (E13-E14)	GACAGATTTCCACCTGACAGC	TGTCTAGCAGCACCTGGATGG
Vps 16(E9-E10)	GTAGCCGTCCTCGGAGCAAGG	GGAACTCATGGGTGCTTCGGG
Dusp22 (E3-E4)	CAAGAGATGCAGAACAGTTGA	GGTTTTGAGATGGTGTGTCTG
Mphoph9 (E3a-E4)	GGGCCACCTGTCATTCA	TCTCAGAAAAGAAGCCCATTTG
Uspl1 (E1-E4)	GGAGTTCGGGTCCACTG	CTTGCCTTTCGCTCTACAAG
Mark2 (E15-E17)	CACAAACCGAAGCAGGAAC	TGCTTCGACTGGACACAC
Agrin (E31-E33)	TGTCCTGGGGGCTTCTCTGG	CGGGAATCCAGAGTTTCGGG
Agrin (E31-E34)	TGTCCTGGGGGCTTCTCTGG	CAACCTTTCCAATCCACAGCACC
Atxn10 (E10-I10)	ATGAGCTGGATGGCATTCCT	GGAAAACTAATGGGCACAGA
Mapk8 (E7-I7)	GGCCAAAGATAAGGATACGA	GAGTCAGCTGGGAAAAGCAC
hnRNPA2/B1(E6-E9)	GGATTCTCGTGGTGGCGG	CATATGGTCCTCCCATGTTC
Parp1 (E21-E23)	TATGAACTCAAGCATGCTTCAC	CTCGCACTTTACCACAGGGATGTC
Harsl (E3-E6)	TCCTCAACAGATGGTTGTGAGGGAGAA	AGTCCCCCAGCTGCAGTCCACTTAGGA
hnRNPD (E1-E3)	CAGAGGGAGCCAAGATCG	TAGGATCTAACTTCAGAGTGC
Tmem41b (E2-E5)	GGATCAGCAAGAACATCACTC	TAGCAGAATGAGGCACCAAGT
Clcn7 (E4-E7)	ACAACAGCGAGAATCAGCTCTTCCTGG	ATTGAGGAAGCACTTGATCTGAGGGAT
Thoc2 (I37)	TCTGGTGGCAAAAAGGAATC	GTCTGTCCACACCCTTGGTT
Gapdh (E3-E5)	ACCACCATGGAGAAGGCCGGG	CAGTGATGGCATGGACTGTGG

Supplementary Figure 1

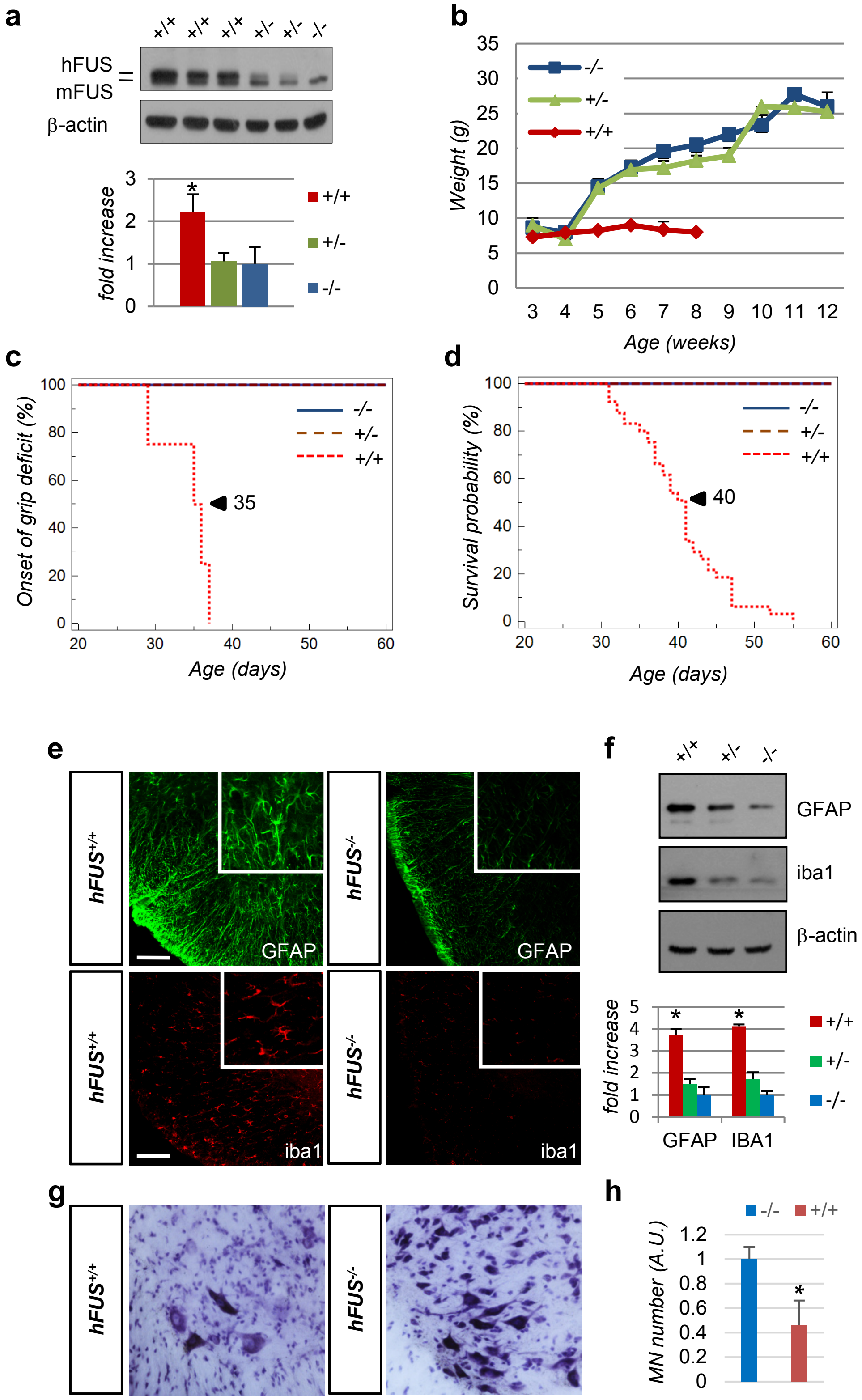

Supplementary Figure 2

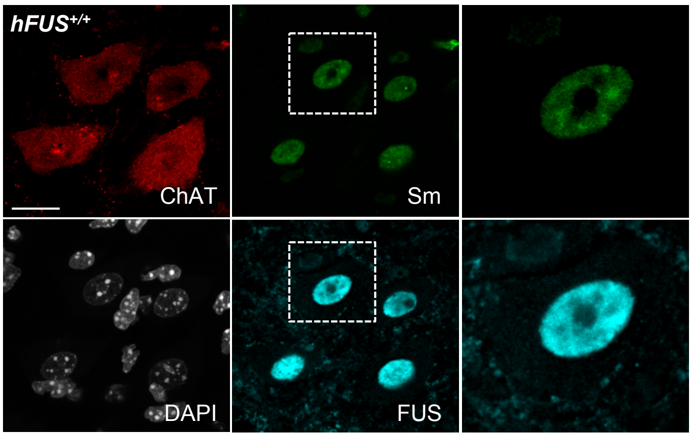

Supplementary Figure 3

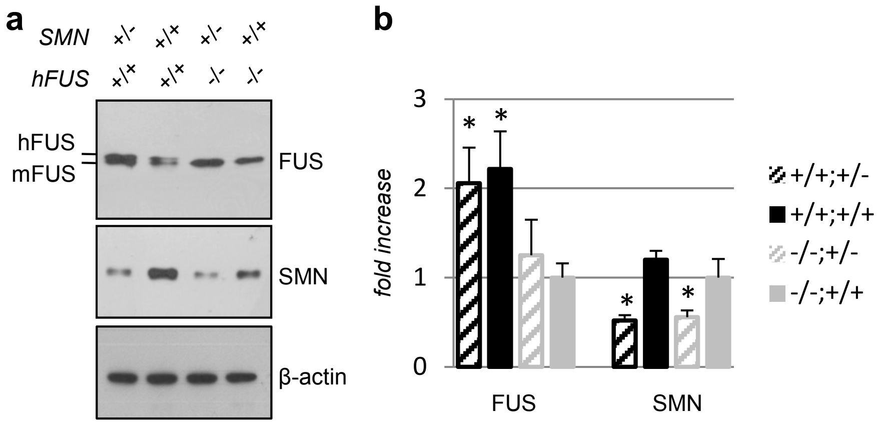

Supplementary Figure 4

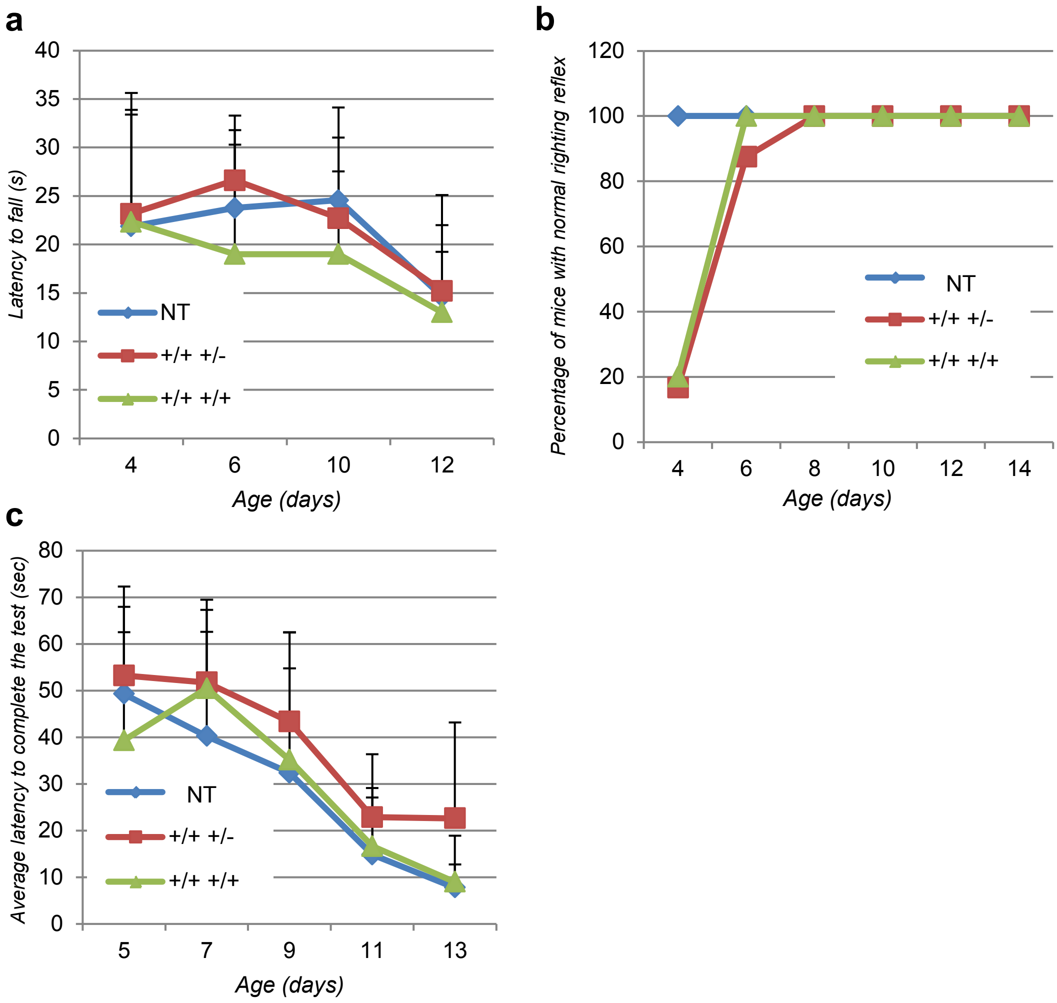

Supplementary Figure 5

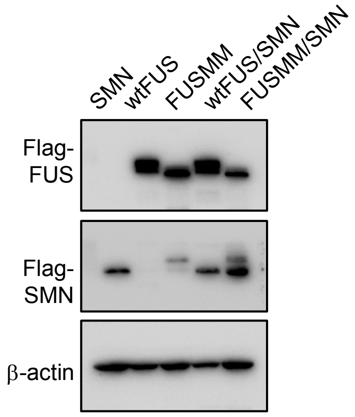

Supplementary Figure 6

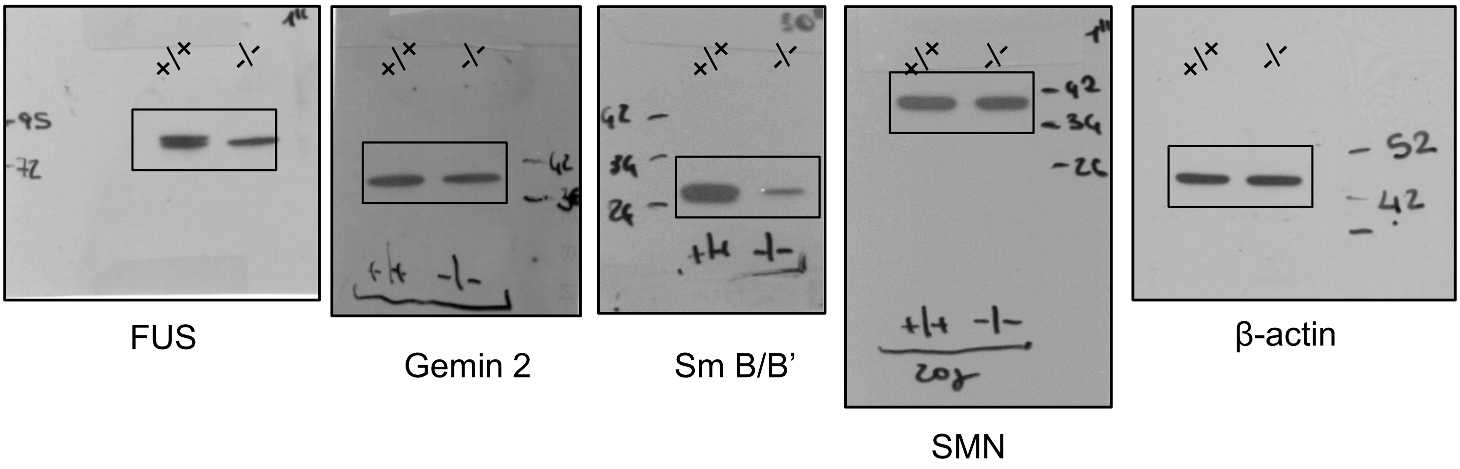

Supplementary Figure 7

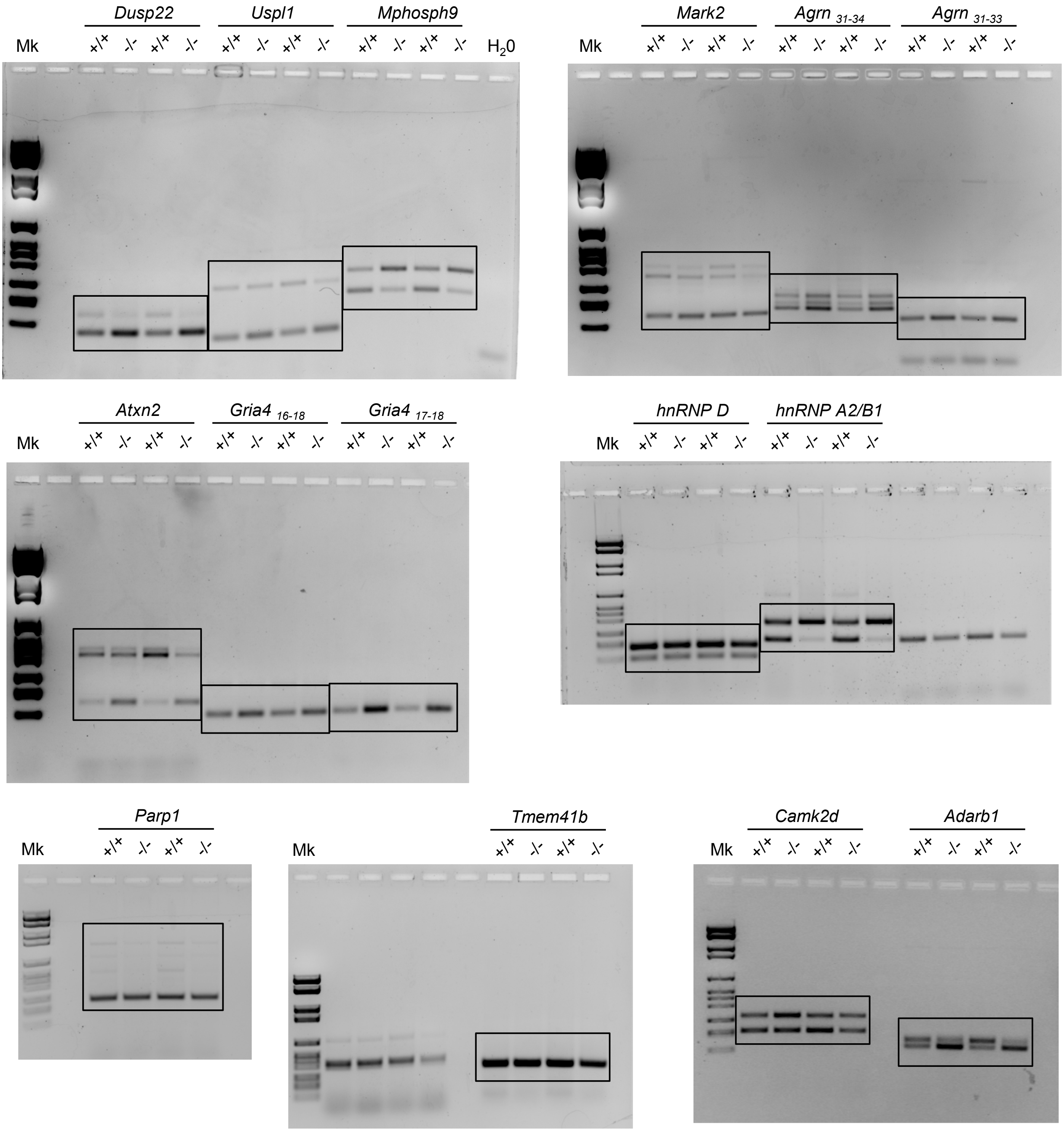

Supplementary Figure 7

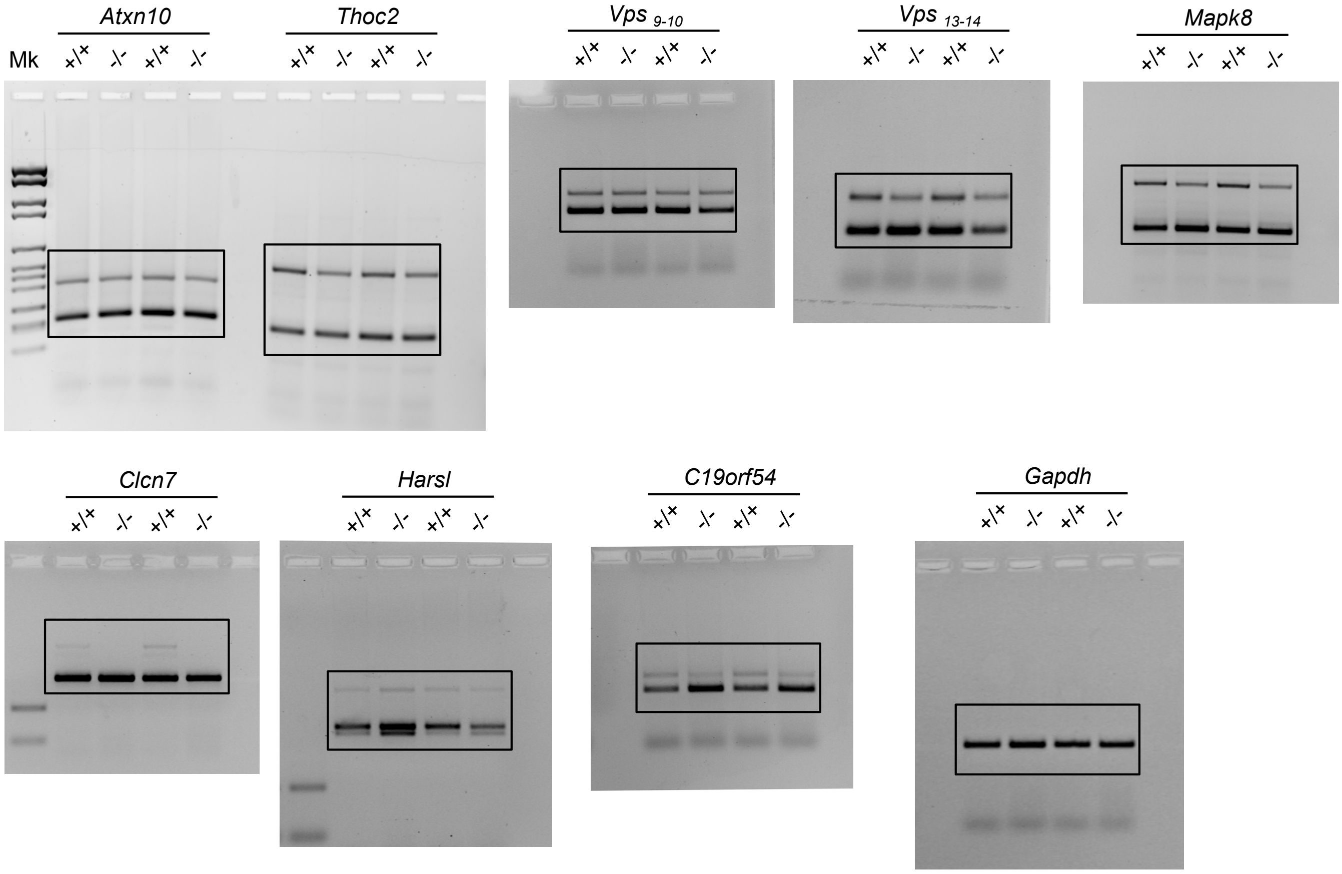

Supplementary Figure 8

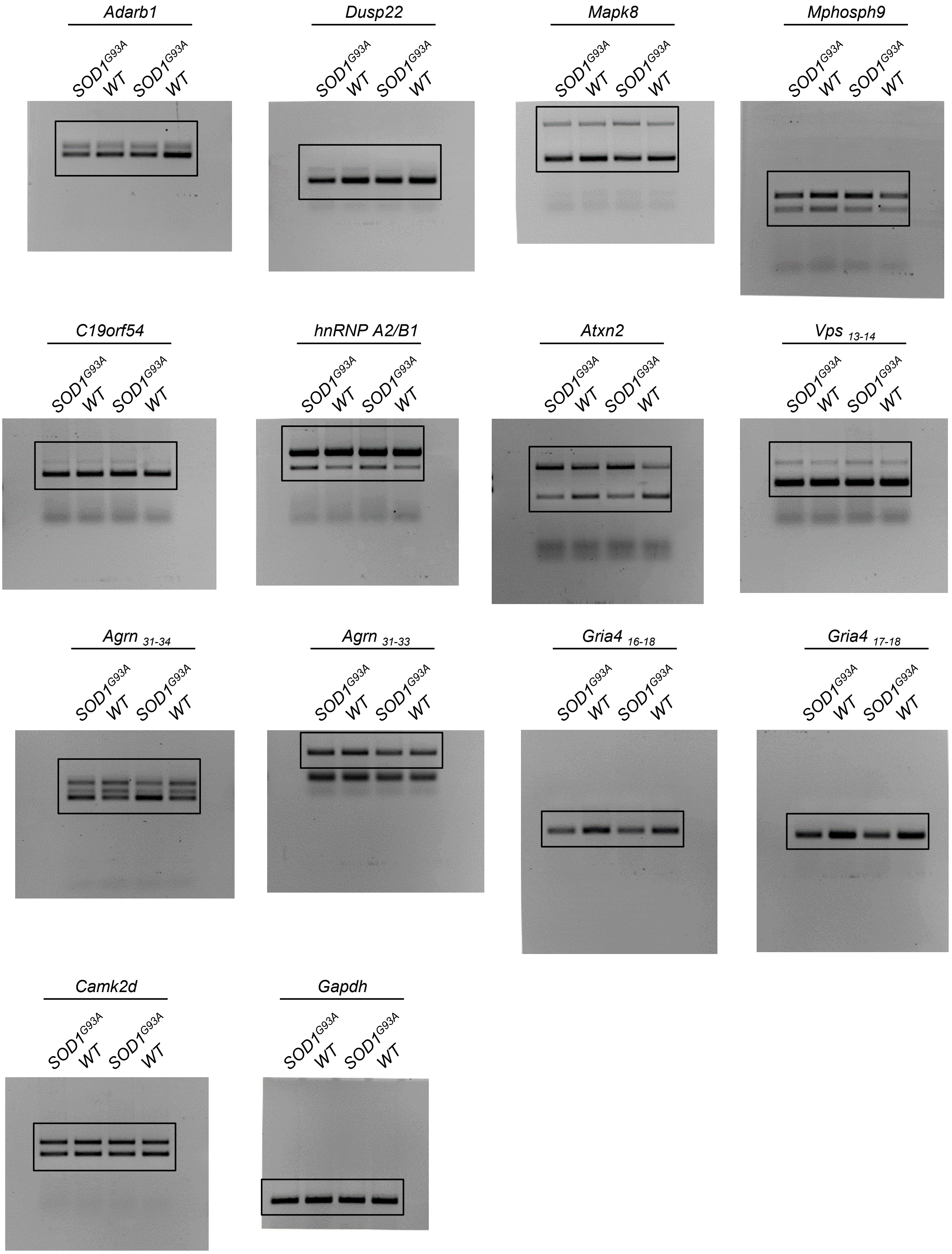

Supplementary Figure 8

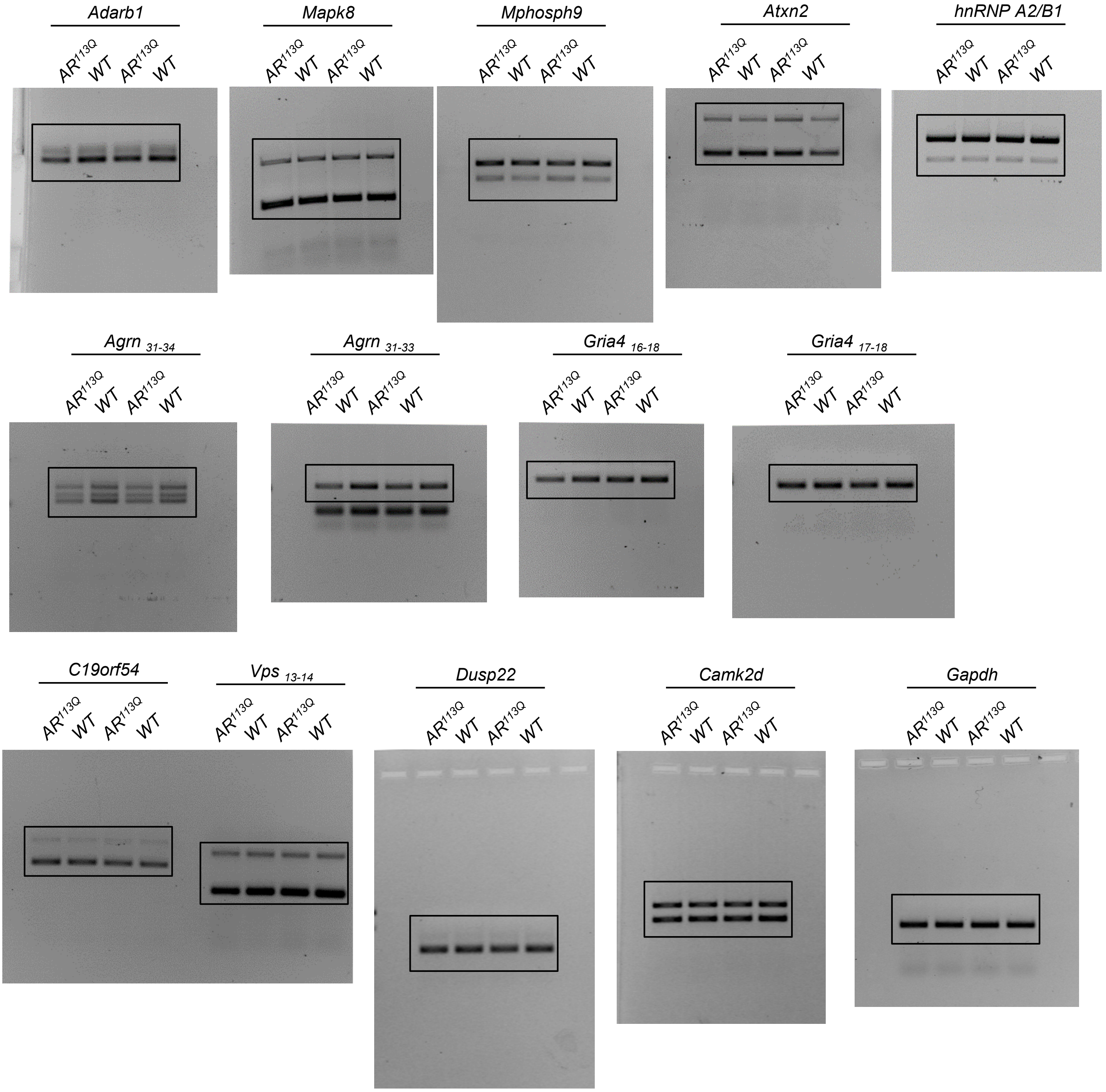

Supplementary Figure 9

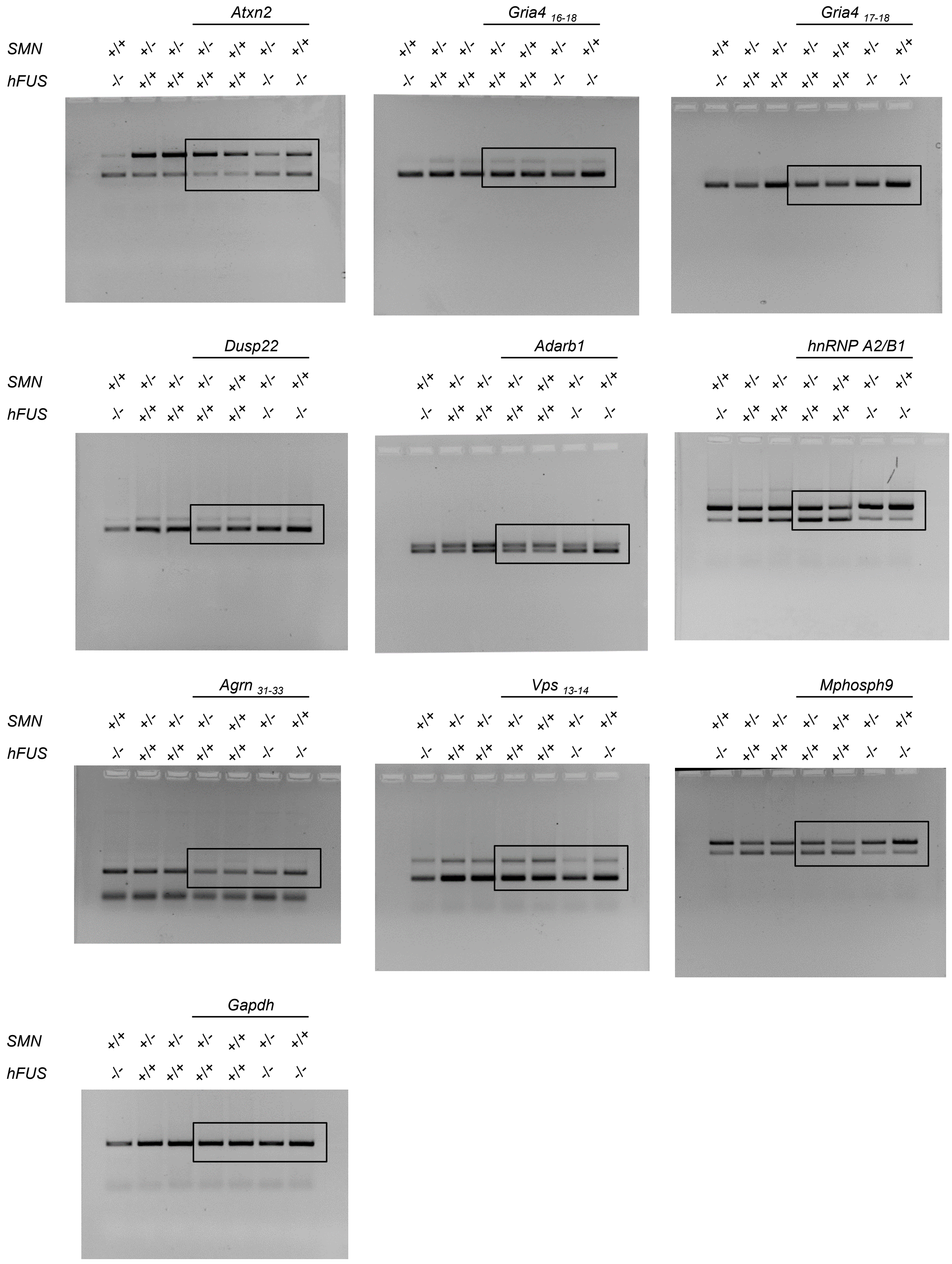

Supplementary Figure 10

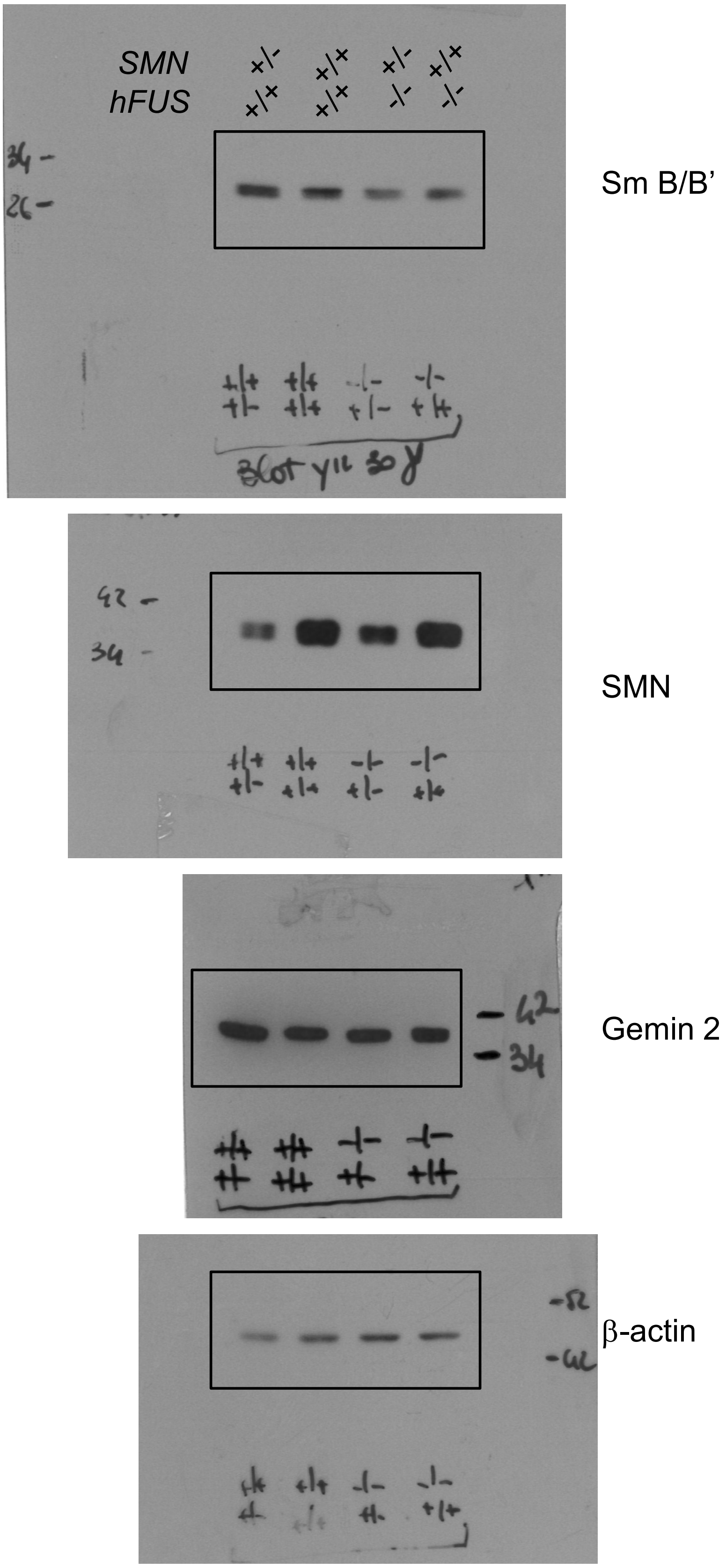

Supplementary Figure 11

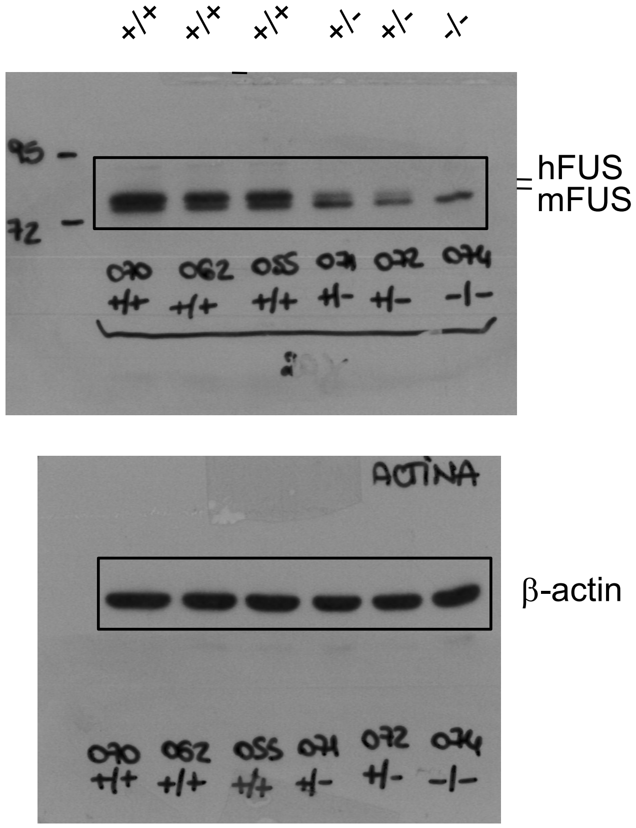

Supplementary Figure 11

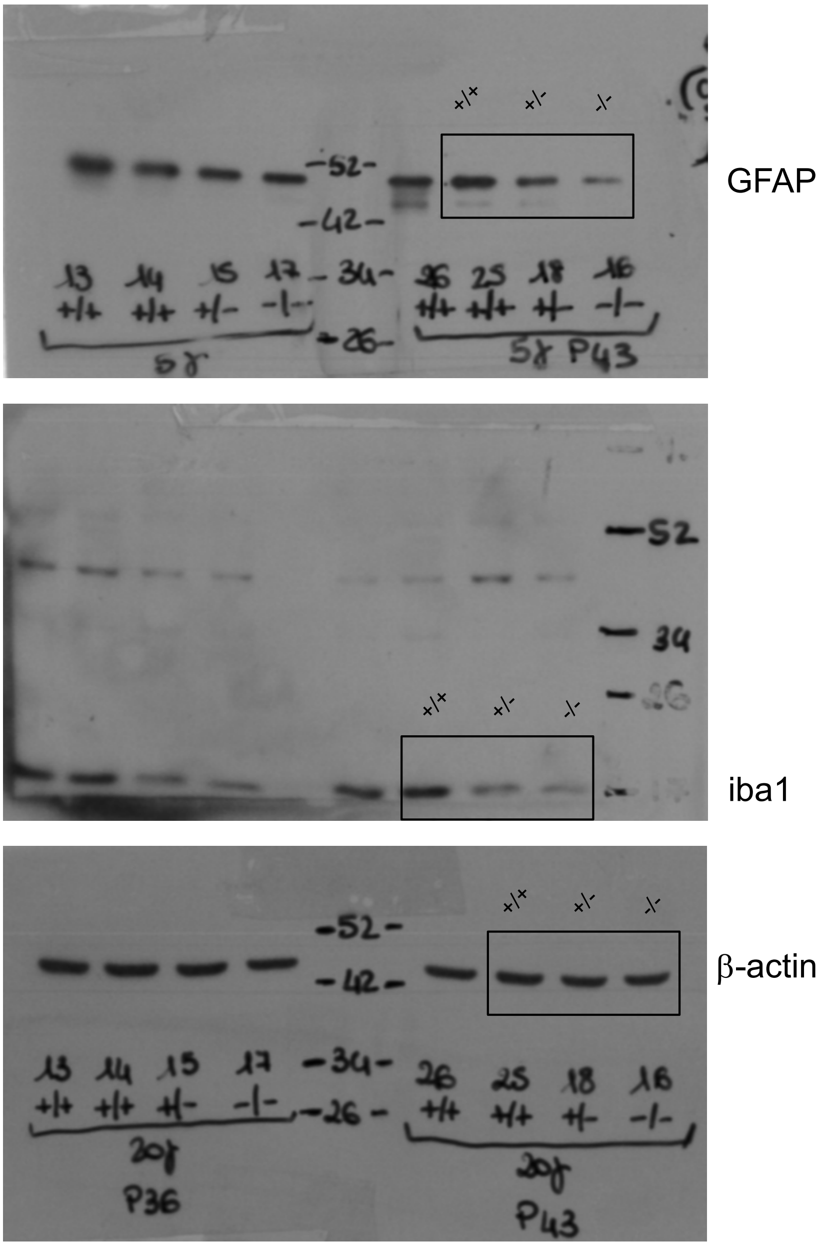

Supplementary Figure 12

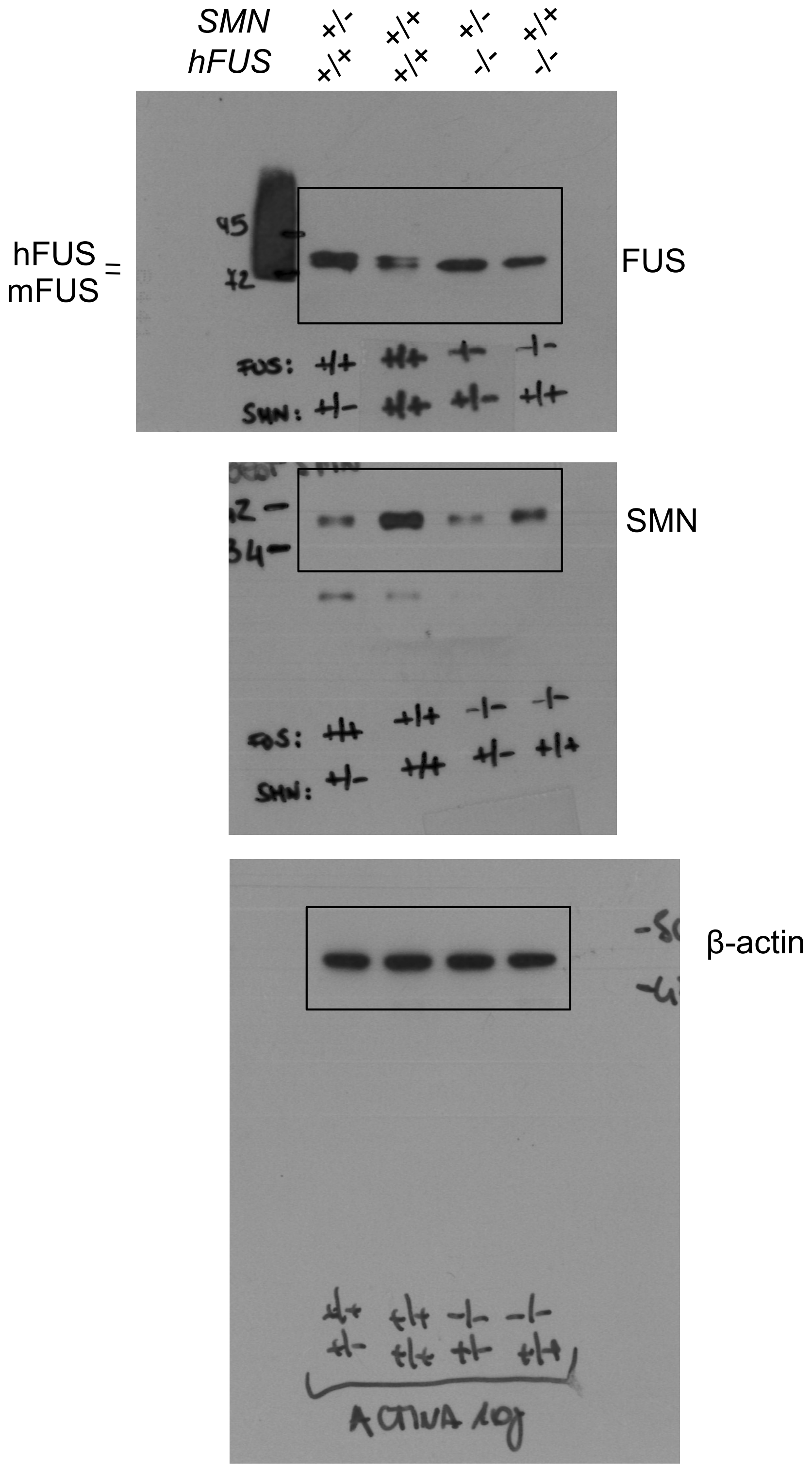

Supplementary Figure 13

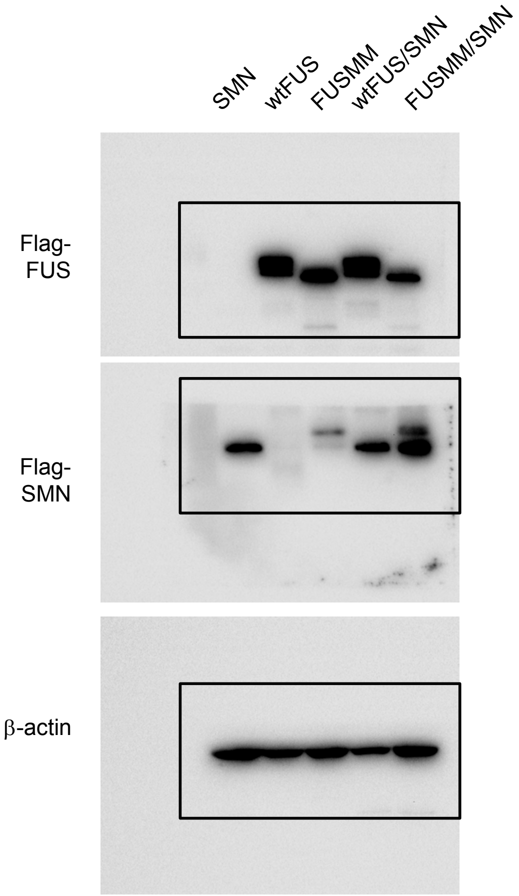

Supplement: Supplementary file 1 — Supplementary Material [file 41598_2017_2195_MOESM1_ESM.pdf]
